# Small RNA profiling for identification of miRNAs involved in regulation of saponins biosynthesis in *Chlorophytum borivilianum*

**DOI:** 10.1186/s12870-017-1214-0

**Published:** 2017-12-28

**Authors:** Monika Kajal, Kashmir Singh

**Affiliations:** Department of Biotechnology, Panjab University, BMS Block-I, Sector 25, Chandigarh, 160014 India

**Keywords:** microRNA, *Chlorophytum borivilianum*, Illumina sequencing, Saponin, Mevalonic acid pathway

## Abstract

**Background:**

MicroRNAs act as molecular regulator of cell signaling, plant growth and development, and regulate various primary and secondary plant metabolic processes. In the present study, deep sequencing of small RNAs was carried out to identify known and novel miRNAs from pharmaceutically important plant, *Chlorophytum borivilianum*.

**Results:**

Total 442 known miRNAs and 5 novel miRNAs were identified from young leaf small RNA library. Experimental validation with stem loop RT-PCR confirmed the in silico identification. Based on transcriptome data of root and leaf of *C. borivilianum, Oryza sativa,* and *Arabidopsis thaliana* target gene prediction was done using psRNAtarget and mirRanda. BLAST2GO helped in localization of predicted targets and KEGG (Kyoto Encyclopedia for Genes and Genomes) pathway analysis concluded that miR9662, miR894, miR172, and miR166 might be involved in regulating saponin biosynthetic pathway. The correlation between miRNA and its target gene was further validated by RT-qPCR analysis.

**Conclusion:**

This study provides first elaborated glimpse of miRNA pool of *C. borivilianum*, which can help to understand the miRNA dependent regulation of saponin biosynthesis and to design further metabolic engineering experiment to enhance their contents in the plant.

**Electronic supplementary material:**

The online version of this article (10.1186/s12870-017-1214-0) contains supplementary material, which is available to authorized users.

## Background

*Chlorophytum borivilianum* Santapau & Fernandes is a monocotyledonous perennial herb of liliaceae family. Genus *Chlorophytum* consists of about 215 species, out which *C. borivilianum, C. arundinaceum Baker, C. tuberosum (Roxb.) Baker, C. laxum R. Br., C. attenuatum Baker, and C. breviscapum Dalz*. are found in India [1, 2]. *C. borivilianum* is a tetraploid species (2n = 4× = 28) with the basic chromosome number 7 [3, 4]. Extracts of this plant possess immunomodulatory [5], anti-diabetic [6], pendiculatory [7], and androgenic activities [8] mainly attributed to high amount of saponins present in the plant [5]. Due to aphrodisiac properties of *C. borivilianum*, this plant is also dubbed as ‘Herbal Viagra’.

Chemically, saponins are classified as triterpenoid and steroidal glycosides. Steroidal saponins are 27 C-atoms molecules, whereas triterpenoid saponins are 30 C-atoms molecules. Saponins consist of non-polar aglycones (triterpene or steroid) and one or more glycone (monosaccharide) moieties [9]. Saponins are excellent emulsifiers and foaming agents because of the presence of both hydrophilic and hydrophobic moieties. These properties might help in lowering the serum cholesterol level in human beings. Saponins act as antioxidant and helps in reduction of free radicals to prevent oxidative stress. Saponins tastes sweet to bitter and posses additional properties like foaming, pharmacological, medicinal, haemolytic, antimicrobial, insecticidal, molluscicidal activities, and find some place in cosmetic industry, beverages, and confectionery [10]. Saponins are present in high amounts in a variety of plant species like *C. borivilianum*, *Glycyrrhiza glabra, Panax ginseng, Bacopa monnieri, Ilex paraguariensis* etc. [11].

In *C. borivilianum,* a number of saponins have been reported, such as furostane type steroidal saponins: Borivilianoside-A (C_56_H_94_O_27_), Borivilianoside-B (C_57_H_96_O_27_), Borivilianoside-C (C_57_H_96_O_28_) and Borivilanoside-D (C_56_H_92_O_27_) [12]. Four spirostane-type steroidal saponins like Borivilianosides-E (C_73_H_120_O_39_Na), Borivilianosides-F (C_73_H_118_O_39_Na), Borivilianosides-G (C_51_H_82_O_24_), Borivilianosides-H (C_50_H_80_O_24_) [13]. Another saponin, Chlorophytoside-I (3b, 5a, 22R, 25R)-26-(β-D-glucopyranosyloxy)-22-hydroxy-furostan-12-one-3ylO-β-D-galactopyranosyl(1-4)lucopyranoside was isolated [14]. Recently, 1′-acetoxychavicol acetate (ACA) was found from *C. borivilianum* roots [15]. Hence, a diverse variety of saponins are present in this plant. Saponins are biosynthesized by mevalonic acid pathway (MVA) in cytoplasm and non-mevalonate pathway (MEP) in plastids. Previously, transcriptome studies of root and leaf of *C. borivilianum* have revealed genes involved in saponin, flavonoid, and alkaloid biosynthetic pathways [16]. Differential expression study of genes coding for enzymes involved in saponins biosynthetic pathway confirmed the activation of early and late-stage genes of the pathway in leaf and root respectively [17–19].

Due to pharmaceutical importance of these phyto-constituents, research is now focusing on to enhance the saponins content in *C. borivilianum*. Increased saponin contents were reported when plantlets were inoculated with mycorrhizal fungi [20]. However, the molecular factors regulating the production of saponins contents are not identified yet. Since the discovery of miRNAs, these are known to regulate gene expression at transcriptional and post transcriptional level. In recent years, miRNAs were found to be major biological factor regulating the secondary metabolite production in many plants [21]. In *Mentha* spp.*,* miR5021 was reported to check the essential oil biosynthesis by regulating expression of genes coding for enzymes such as geranyl di-phosphate synthase involved in 2-C-methyl-D-erythritol 4-phosphate/1-deoxy-D-xylulose 5-phosphate (DOXP) pathway [22]. miR5021 and miR5293 were found to regulate first enzymatic function of the MVA pathway in *Panax notoginseng* [23]*.* Based on the above studies, we hypothesized that there must be some miRNAs regulating the secondary metabolic pathway in *C. borivilianum*. Our aim was to profile miRNAs in *C. borivilianum* and to predict targets of the identified miRNAs. Further involvement of miRNAs targets in biochemical pathway were studied, with special emphasis on saponins biosynthetic pathway. To achieve this, we prepared small RNA library and sequenced it using Illumina platform. Known and novel miRNAs were predicted along with their gene targets. RT-qPCR analysis was performed to establish a correlation between miRNAs and their corresponding targets predicted by computational analysis.

## Methods

### Plant material

*C. borivilianum* plants were raised from vegetative buds of old plants in the soil:soilrite (1:1.5) mix under regulated environment in plant growth chamber with 27 °C day/night temperature, illuminated with white light (flux density 200 μmolm^−2^ s^−1^) at Panjab University, Chandigarh (India) [latitude: 30° 44′14 N; longitude 76°47′14E; altitude 350 m above mean sea level]. Young leaves were collected from the 2 month old plant for isolation of total RNA enriched with small RNA.

### RNA isolation & small RNA sequencing

Extraction of total RNA including small RNA was carried out by combining the Ghawana et al. 2011 [24] protocol with miRNeasy kit (Qiagen, Germany). Fine powder of young leaf tissue (100 mg) was homogenized with solution-I and solution-II. Mixture was then treated with 200 μl chloroform to separate the organic phase. Upper aqueous phase was then separated, and 1.5 volume of 100% ethanol was added. The solution was loaded onto miRNeasy column for washing and DNase digestion. After on-column DNase digestion and washing, total RNA enriched with small RNA was eluted using nuclease free water. Further, RNA quantity and quality were examined using Nanodrop spectrophotometer and Agilent 2100 bioanalyzer system respectively. Small RNA sequencing was performed using Illumina HiSeq 2000 Platform at AgriGenome Labs Private limited, Smart City Kochi, Infopark Road, Kakanad, Kerala, India (http://www.aggenome.com/).

Raw data of small RNA sequencing was submitted to SRA (Sequence Read Archive) database of NCBI (Natioanl Centre for Biotechnology Information). Small RNA reads (50 bp length) were generated using Illumina platform. Low quality reads were removed on the basis of GC content, average base quality, and phred score. Raw reads were then processed and 5′ and 3′ adapter sequences were eliminated using Cutadapt tool (v-1.3) [25]. Remaining reads were then aligned against a number of databases like GtRNAdb, Rfam, piRNABank, siRNAdb, NCBI Genbank, deepBase to eliminate other non-coding RNAs like tRNA, rRNA, piRNA, siRNA, snRNA, and snoRNA, respectively using Bowtie2 program (version 2.1.0) [26]. The remaining reads were used to predict known and novel miRNAs.

### Identification of known and novel miRNAs

The filtered small RNA reads (17-35 bp) were used for known miRNA identification. These reads were aligned with mature miRNAs and precursor sequences of viridiplantae dataset in miRBase-21 [27] using Bowtie program (version 0.12.9) allowing only 2 mismatches with mature miRNAs [28].

Unaligned reads left after known miRNAs prediction were taken further for novel miRNAs prediction using miRDeep2 (version 2.0.0.7) [29]. Reads were aligned to leaf and root transcriptome sequences of *C. borivilianum.* These reads were checked for hairpin loop formation using RNA folding form (version 2.3 energies) application from Mfold webserver [30]. miRNA and miRNA* sequences were found in the arm of the stem-loop. Ultimately, these reads were considered as the precursor of novel miRNAs. Sequences with the following characteristics can be considered as potential precursor of novel miRNAs: (a) sequence forming stem-loop hairpin structure (b) sequence of mature miRNA within one arm of hairpin structure (c) less than 6 mismatches are allowed between miRNA and miRNA* (d) secondary structure with minimum fold free energy lower than or equal to -18 kcal/mol (e) AU% within the range of 30-70% [31].

### Validation of miRNA using stem-loop RT-PCR

Randomly chosen 5 known miRNAs and all 5 novel miRNAs were selected for validation. miRNA-specific stem-loop RT-primers for reverse transcription were designed according to Kramer, 2011 [32] (Additional file 1). cDNA was prepared for each miRNA (iScript™ Select cDNA Synthesis Kit, Biorad) and PCR amplification of each miRNA was carried out using miRNA specific forward primer and universal reverse primer. PCR product was eluted, ligated into pGEM®-T easy vector and ligated product was transformed to competent *E. coli* DH5α cells. Recombinant plasmids were isolated, sequenced and analyzed.

### Target prediction for identified miRNAs

In silico target gene prediction of known and novel miRNAs in *C. borivilianum* was done using target gene prediction softwares, psRNATarget [33] and miRanda [34]. Identified miRNAs were targeted against root and leaf transcriptome library of *C. borivilianum*, *Oryza sativa* (rice, transcript, TIGR (The Institute for Genomic Research) genome cDNA OSA1 Release 5 (OSA1R5), version 5) and *Arabidopsis thaliana* (transcripts, removed miRNA gene, TAIR (The Arabidopsis Information Resource) version 10, released on 2010_12_14) using psRNATargetV2 (2017 release) with default parameters [maximum expectation: 3; length for complementarities scoring (hspsize): 19; target accessibility–maximum energy to unpair the target site (UPE): 25; flanking length around target site for target accessibility analysis: 17 bp (upstream)/13 bp (downstream); range of central mismatch leading to translational inhibition: 10-11 nt]. It is likely that psRNATarget allocates more than one target for each miRNA sequence and multiple target sites of a particular mRNA molecule because it takes into account both complementarities and site accessibility of the targets. Once potential target mRNA sequences were obtained, target transcripts were subjected to BLASTX (Basic Local Alignment Search Tool) search against nr (Non Redundant) database from NCBI to predict function of potential targets. To further see the role of miRNAs in saponin biosynthetic pathway, mature known and novel miRNAs were targeted specifically against the transcripts coding for enzymes involved in MVA and MEP pathway using miRanda. The prediction was made using miRanda with detailed statistical study of minimum free energies (MFEs) using default parameters [Gap open penalty: −0.9; Gap extend: −0.4; Score threshold: 50.0; Energy default: −0.20 Kcal/mol].

Finally for all identified targets, Gene Ontology (GO) annotations were retrieved using Blast2GO 4.1 software at Biological Process (BP), Molecular Function (MF), and Cellular Component (CC) levels [35]. Finally, identified targets were located in biochemical pathways by Kyoto Encyclopedia of Genes and Genomes (KEGG) pathway analysis.

### RT-qPCR based expression analysis of miRNAs and their predicted targets

To confirm relationship of miRNAs and their targets, leaf tissue of *C. borivilianum* at two stages were collected. One, at the young stage (2 month old) and another at dormancy stage (7 month old). Total 11 conserved miRNAs were selected that were computationally predicted to regulate the genes coding for enzymes involved in saponins biosynthesis. Primers for stem-loop RT-qPCR of conserved miRNAs were designed as described previously [32], and the primers for target genes were designed using Primer 3.0 input software (Additional file 1).

Total RNA along with small RNA isolation [24] and cDNA preparation was done according to protocol described earlier. Reaction cocktail for RT-qPCR of miRNAs and their targets were prepared using SsoFast™ Evagreen® Supermix and the experiment was performed using Bio-Rad CFX96™ Real-Time PCR system. β-actin was used as an internal control. 1 μl of each cDNA sample was used for analysis along with 10 μl of SYBER green, 7 μl of nuclease free water, 1 μl of each miRNA specific forward primer and stem loop complimentary universal reverse primer in cycling condition of hot start at 95 °C for 2 min, 45 cycles of denaturation at 95 °C for 15 s, annealing at 55 °C for 30 s and extension at 72 °C for 30 s. All experiments were run in triplicates. To finally calculate the fold change expression of miRNAs and their targets **ΔΔ**C_T_ method was used [36]. 2^(− **ΔΔ**C_T_) values were changed to (log_2_) to generate fold change expression.

## Results

### Raw data analysis

Total 79,419,700 raw reads (1 × 50 bp) were generated using Illumina Hiseq 2000 platform. After the removal of low quality reads, short fragments, and adaptor sequences, a total of 22,155,316 clean reads were obtained (Table 1). Alignment of clean reads with various databases identified sequences of other non-coding RNAs like siRNAs (0.51%), piRNAs (3.10%), snRNA (0.02%), snoRNA (0.04%), tRNA (0.52%), and rRNA (11.30%). Then remaining 18,723,614 (84.51%) reads were filtered, out of which 18,057,758 reads of length 17-35 bp were used for the identification of known miRNAs.

**Table 1:** Summary of Illumina sequencing data for small RNAs of *C. borivilianum*

Type	Number of Reads	%	Number of Unique IDs
Total sRNA sequenced in library	7,94,19,700		
Number of preprocessed reads(Read length greater than 4 bp taken for further analysis)	2,21,55,316	100	
Number of reads aligned to siRNA	1,12,261	0.51	627
Number of reads aligned to piRNA	6,86,934	3.1	1,24,864
Number of reads aligned to snRNA	4538	0.02	273
Number of reads aligned to snoRNA	9285	0.04	973
Number of reads aligned to tRNA	11,565	0.52	41,945
Number of reads aligned to rRNA	25,03,033	11.3	5,87,798
Number of reads unaligned after contamination removal (4 bp-50 bp)	1,87,23,614	84.51	
Number of reads with the length of 17 bp- 35 bp	1,80,57,758		

### Known miRNA identification

**Fig. 1:**
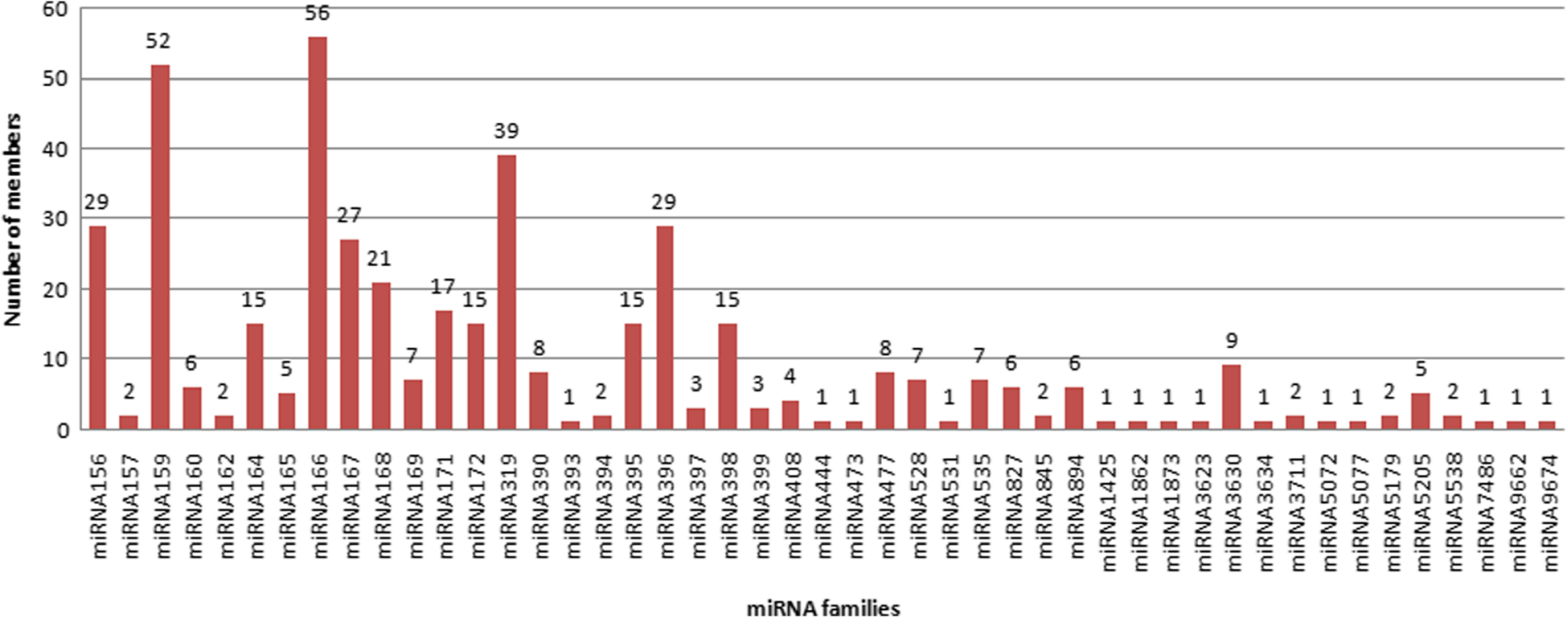
Number of known miRNAs in each miRNA family in *C. borivilianum*

**Fig. 2:**
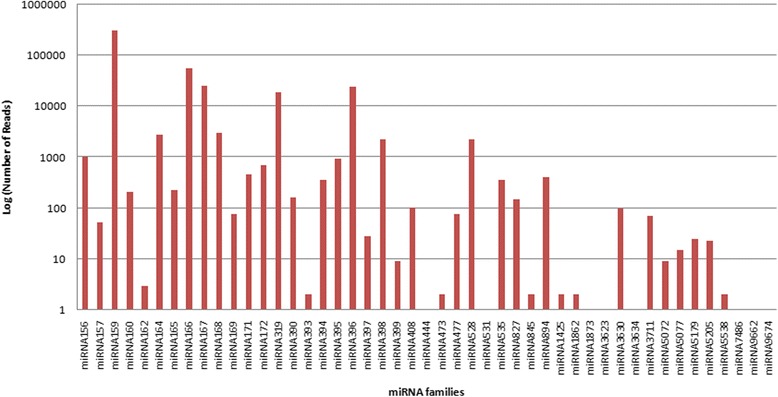
Abundance of *C. borivilianum* miRNA families

Total 442 known miRNAs belonging to 47 miRNA families were identified from leaf’s small RNA population of *C. borivilianum* (Additional file 2). Nomenclature of distinct mature miRNAs originated from same precursor were differentiated by adding a number suffix [37, 38]. By this method, miRNA species diced from a common precursor can be differentiated. During our research, a numbers of homologs were found for each miRNA, miRNAs having more than one homolog but similar sequence were considered in same family. miR166 family with 56 member followed by miR159 (52 members) family were found to have maximum number of members in the library, although 14 miRNA families such as miR393, miR444, miR473, miR531, miR1425, miR1862, miR1873, miR3623, miR3634, miR5072, miR5077, miR7486, miR9662, and miR9674 were found to have only one member. Members of same miRNA family do not reflect the degree of nucleotide (nt) sequence similarity, but the functional equivalence i.e. to have shared functions. Number of members in each family are mentioned in Fig. 1. Total read count for each miRNA was observed, which provided an idea about apparent expression level of miRNA in *C. borivilianum* young leaf tissue. Out of all, on the basis of bioinformatic analysis maximum expression was observed for miR159 family i.e. 315,441 reads followed by miR166 and miR167 family with 56,445 and 25,592 reads respectively and 17 miRNA families were reported to have less than 10 reads. This wide range of expression of different miRNAs predicts that different miRNAs play distinct role in plant growth and development. Graphical representation of abundance of each miRNA family is mentioned in Fig. 2. In *C. borivilianum,* miRNA length was found in the range of 18-24 nt, miRNAs containing 21 nt were the most abundant (31.45%), followed by miRNAs of length 20 nt (Additional file 3).

### Novel miRNA identification

**Fig. 3:**
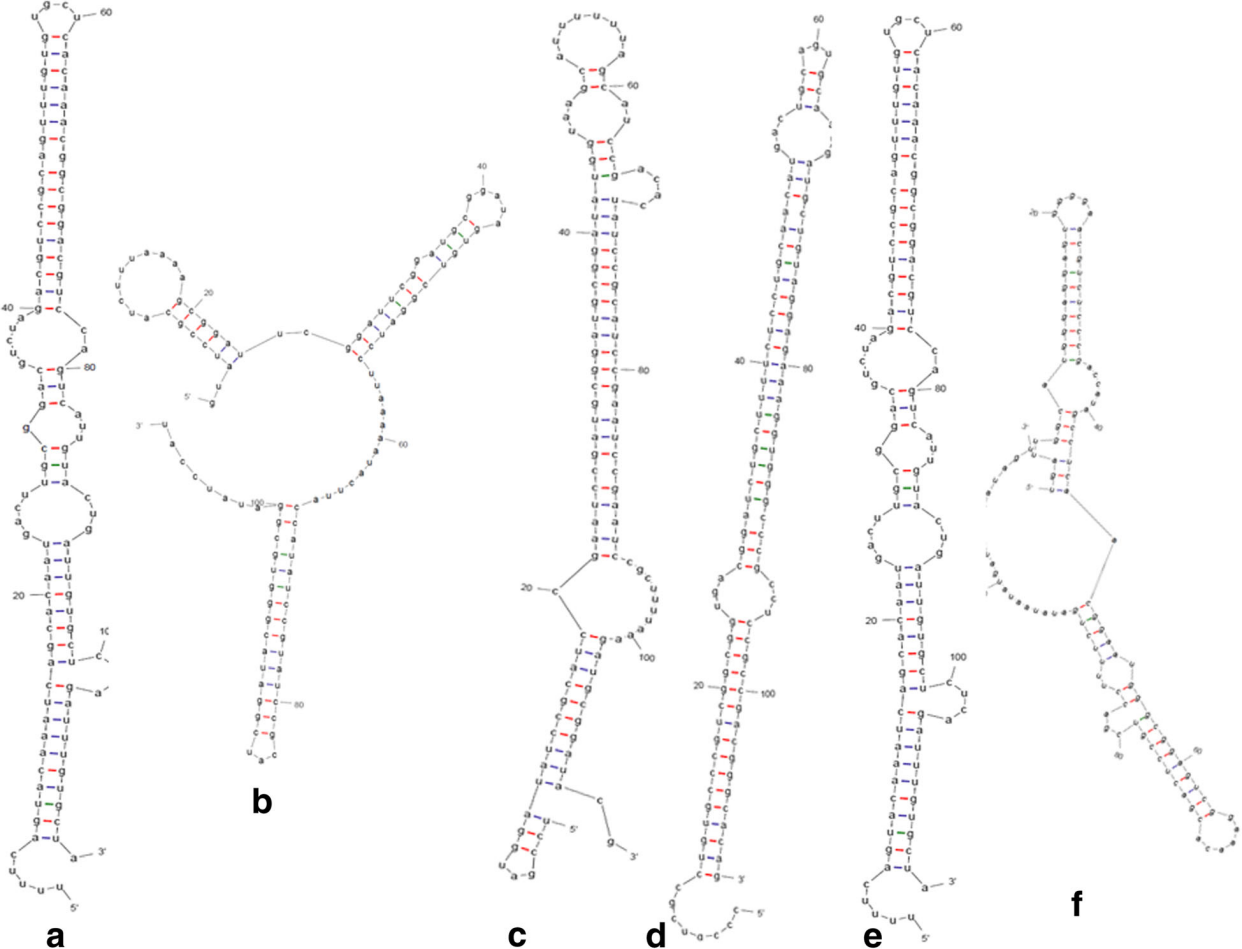
Secondary structures of precursors of novel miRNAs using Mfold web server. **a** cbo-miR1 (leaf) (**b**) cbo-miR1 (root) (**c**) cbo-miR2 (**d**) cbo-miR3 (**e**) cbo-miR4 (**f**) cbo-miR5

Due to unavailability of whole genome sequence of *C. borivilianum,* root and leaf transcriptome data was used as reference for prediction of novel miRNAs. In total, 5 novel miRNAs were scrutinized (Table 2), 2 were aligned with leaf transcriptome and 3 with root transcriptome. Interestingly one miRNA i.e. cbo-miR1 was observed to have different precursor in leaf and root with same level of expression. This suggests that cbo-miR1 functions in both root and leaf but have discrete origin. Identified novel miRNAs were ranged from 21 to 24 nt. miRNA cbo-miR3 was found to be most abundant. Variation in the abundance of plant specific miRNAs was observed in a wide range i.e. from 28 reads for cbo-miR2 to 23,252 reads for cbo-miR3. Contrary to many previous studies, sequence analysis of novel miRNAs indicated that none of them started with 5’U. Ability of miRNA precursor to form a stem-loop can confirm its biosynthesis of mature miRNA. For that precursors of predicted novel miRNAs were used for secondary structure formation using RNA folding form (version 2.3 energies) from Mfold web server. This gives dG (free energy) for each folding structure. Secondary structures of all 5 novel miRNAs are described in Fig. 3. cbo-miR3 (dG = −63.60 kcal/mol at 37 °C) was found to be the most stable novel miRNA on the basis of free energy and cbo-miR5 (dG = −35.70 kcal/mol at 37 °C) with minimum stability in *C. borivilianum*. Stem-loop RT-PCR was carried out to confirm the in silico identified known and novel miRNAs. Cloning of miRNAs was done and sequencing of recombinant plasmids verified the computationally identified miRNAs (Additional file 4). In silico analysis followed by miRNA cloning and sequencing confirmed that novel miRNAs were indeed part of miRNAs family of *C. borivilanum*.

**Table 2:** Novel miRNAs identified small RNA population of C. borivilianum

miRNA	Novel Mature Sequence	Length	Read Count	AU(%)	Strand	MFE for Precursor
				Precursor		
cbo-miR1	AAUGACUUGCGGACGUCUAGACGU	24	4268	48.7	+	−34.14
cbo-miR2	AUCCGCAUCCGAAUCCGAAUCCGC	24	28	49.3	+	−29.15
cbo-miR3	GCGGUGACGGAUCUGCUUUUC	21	23,252	40.7	–	−34.14
cbo-miR4	AAAAGCGGAUUCGGAUUCGGAUGC	24	469	53.1	–	−34.14
cbo-miR5	CGACUCCGUCGACCUUUUCUGA	22	5194	43.2	–	−29.15

### Target prediction and gene ontology

To better understand the function of identified miRNAs in *C. borivilianum*, target prediction of known and novel miRNAs was carried out. To annotate the potential targets, BLASTX search against nr (Non Redundant) database was performed. These targets were annotated as transcription factors like GAMYB (Gibberellin and Abscisic acid-regulated MYB) transcription factor, Auxin Response Factor (ARF) family proteins, Growth regulating factors, D-tyrosyl-tRNA(Tyr)deacylase, Squamosa promoter-binding-like (SPL) protein, UDP-glucuronate decarboxylase, leucine-rich repeat receptor-like serine/threonine-protein kinase, phosphoenolpyruvate phosphatase-like, protein HUA ENHANCER 2-LIKE 1. Vast variety of targets were identified suggesting role of miRNAs in regulating key functions of the plant such as non-coding RNA biogenesis, disease resistance, signal transduction, and stress responses (Additional file 5). A parallel target prediction of miRNAs was carried out using transcriptomic data of *O. sativa* and *A. thaliana*. For this, targets of known miRNAs were also found in *O. sativa* transcriptome, and *A. thaliana* transcriptome. Description of targets from *O. sativa* and *A. thaliana* are stated in Additional files 6 and 7 respectively. Secondary metabolites are mainly responsible for extensive medicinal properties of *C. borivilianum.* So, it was important to focus on the miRNAs targeting mRNAs involved in saponin biosynthetic pathway. After target prediction for both known and novel miRNAs using miRanda, transcripts were subjected to BLASTX analysis to find the functional proteins. From root and leaf transcriptome, targets were found to be involved in saponin biosynthesis. Target genes code for squalene epoxidase, squalene synthase, squalene monooxygenase, mevalonate diphosphate decarboxylase, mevalonate kinase, phosphomevalonate kinase, chloroplast 1-deoxy-d-xylulose-5-phosphate synthase, geranyl diphosphate synthase, diphosphomevalonate decarboxylase, cycloartenol synthase, cytochrome p450-90b1-like, 4-hydroxy-3-methylbut-2-enyl diphosphate reductase, oxidosqualene cyclase, farnesyl pyrophosphate synthase, UDP-glycosyltransferase family proteins, furostanol glycoside 26-O-beta-glucosidase, isopentenyl diphosphate isomerase 2, hydroxymethylglutaryl-CoA synthase, and 4-hydroxy-3-methylbut-2-en-1-yl diphosphate synthase.

Additionally, novel miRNAs were also found to regulate important biological processes. Total 7 targets for 4 novel miRNAs from leaf and root transcriptome of *C. borivilianum* were found. Cbo-miR3 regulates the expression of genes coding for transketolase, cytochrome P450 family proteins, and epoxide hydrolase A. And novel miRNAs cbo-miR4 and cbo-miR5 target the transcripts coding for cytochrome P450 family protein, glutathione S-transferase-1 respectively, and cbo-miR1 targets beta-glucosidase 12-like isoforms. This suggests, miRNAs with their targets directly involved in the saponin biosynthesis are described in Additional file 8.

**Fig. 4:**
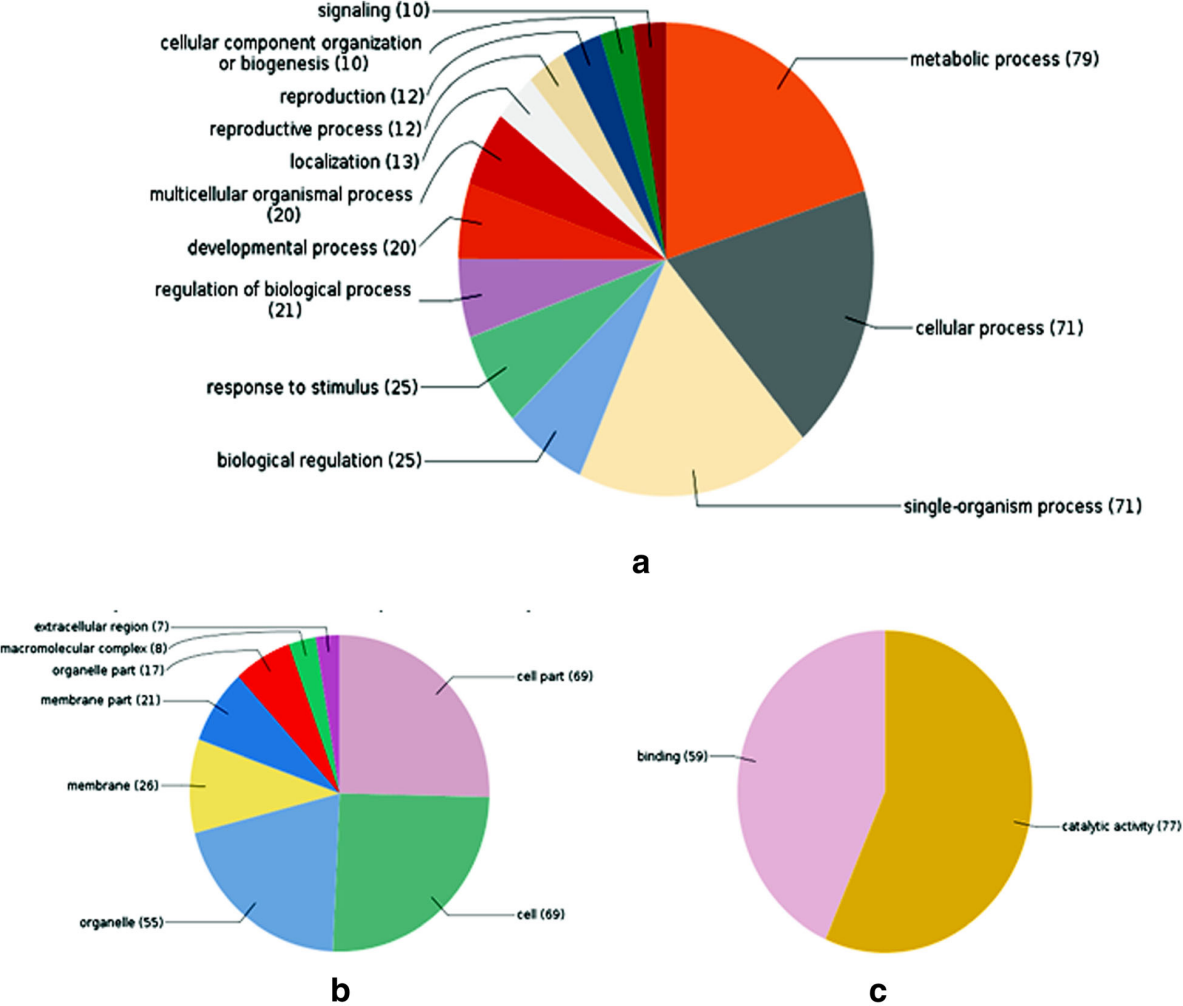
Categorization of target genes according to (**a**) Biological Process (**b**) Cellular Component (**c**) Molecular Function

Gene functional annotation was performed by Blast2GO 4.1 software. Here we categorized the targets on the basis of their involvement in cellular process, metabolic process, and biological role. Total 135 targets predicted by psRNATarget and miRanda were subjected to BLASTX search, mapping, and annotation to analyze and categorize using GO analysis (Fig. 4).

**Fig. 5:**
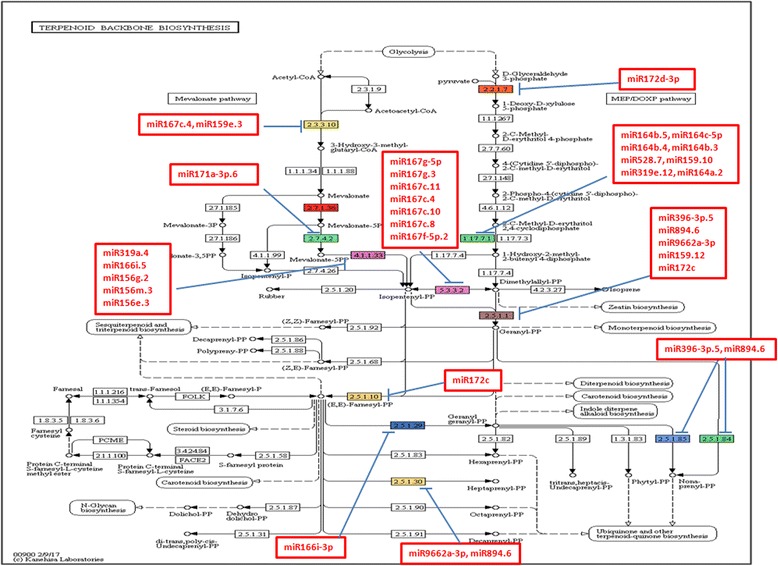
Terpenoid backbone biosynthesis indicating the location of miRNAs and their target genes, EC: 2.5.1.85-all-trans-nonaprenyl-diphosphate synthase, EC: 2.5.1.84-all-trans-nonaprenyl-diphosphate synthase, EC: 2.5.1.1-farnesyl diphosphate synthase and geranylgeranyl diphosphate synthase, EC: 2.3.3.10-hydroxymethylglutaryl-CoA synthase, EC: 5.3.3.2-isopentenyl-diphosphate Delta-isomerase, EC: 2.5.1.10-farnesyl diphosphate synthase and geranylgeranyl diphosphate synthase, EC: 2.2.1.7-1-deoxy-D-xylulose-5-phosphate synthase, EC: 2.7.4.2-phosphomevalonate kinase, EC: 4.1.1.33-diphosphomevalonate decarboxylase, EC: 2.7.1.36-mevalonate kinase, EC: 2.5.1.29-geranylgeranyl diphosphate synthase, EC: 2.5.1.30-heptaprenyl diphosphate synthase, EC: 1.17.7.1-(E)-4-hydroxy-3-methylbut-2-enyl-diphosphate synthase

**Fig. 6:**
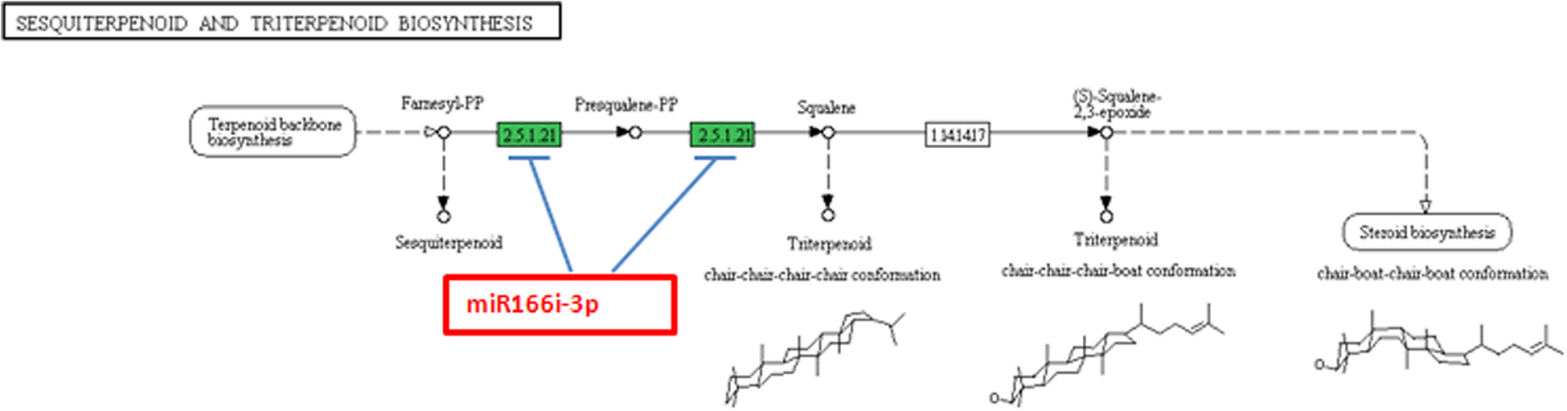
Sesquiterpenoid and Triterpenoid biosynthesis pathway with the target genes location for miRNA; miR166i-3p, EC: 2.5.1.21 – *Squalene synthase*

KEGG pathway analysis revealed total 135 targets in *C. borivilianum* transcriptome are involved in 43 metabolic networks including starch and sucrose metabolism, cysteine and methionine metabolism, galactose metabolism, arachidonic acid metabolism, terpenoid backbone biosynthesis, steroid biosynthesis, butanoate metabolism, sesquiterpenoid and triterpenoid biosynthesis, and pentose phosphate pathway. Total 29 miRNAs (miR172d-3p, miR164b.5, miR164c-5p, miR164b.4, miR164b.3, miR528.7, miR159.10, miR319e.12, miR164a.2, miR396-3p.5, miR894.6, miR9662a-3p, miR159.12, miR172c, miR167g-5p, miR167g.3, miR167c.11, miR167c.4, miR167c.10, miR167c.8, miR167f-5p.2, miR159e.3, miR171a-3p.6, miR319a.4, miR166i.5, miR156g.2, miR156m.3, miR156e.3, miR166i-3p) were found to regulate a single pathway i.e. terpenoid backbone biosynthesis (Fig. 5). Whereas, a single miRNA (miR166i-3p) was found to regulate sesquiterpenoid and triterpenoid biosynthesis (Fig. 6). Predicted KEGG pathways provide information about the functions performed by the miRNAs targets.

### Relative expression profiling of miRNAs and their targets

**Fig. 7:**
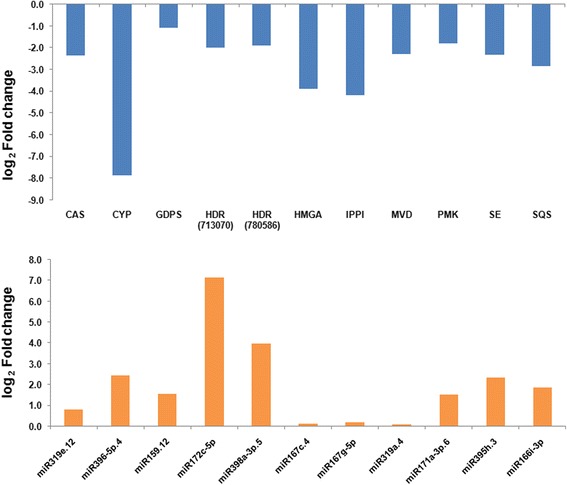
Relative expression analysis of selected miRNAs and their targets involved in saponin biosynthesis by stem-loop qRT-PCR. [CAS: Cycloartenol synthase, CYP: Cytochrome p450 90b1-like, GDPS: Geranyl diphosphat synthase, HDR: 4-Hydroxy-3-methylbut-2-enyl diphosphate reductase, HMGA: Hydroxymethylglutaryl-CoA synthase, IPPI: Isopentenyl diphosphate isomerase, MVD: Mevalonate diphosphate decarboxylase, PMK: Phosphomevalonate kinase, SE: Squalene epoxidase, SQS: Squalene synthase]

To study the association of miRNAs and their corresponding target genes, expression pattern of 11 selected target genes of conserved miRNAs were validated by RT-qPCR. The list of genes along with their targeting miRNAs is mentioned in Table 3. Relative expression study in leaf tissue of young plant and the mature plant reaching dormancy stage was carried out. Young plant was considered as reference to calculate fold change expression. It was observed that the genes involved in saponin biosynthesis were having lower expression during dormancy than active growth period. Simultaneously, the miRNAs expression pattern was observed just opposite to that of targets. The expression of *Cytochrome p450 90b1-like* (*CYP*) was down-regulated by 7.9 log fold while its corresponding miRNA (miR396e-5p.4) was up-regulated by 1.5 log fold. On the same pattern log fold down-regulation of gene coding for a key enzyme of MVA pathway that is Squalene synthase (SQS) was by 2.8 and the comparative up-regulation of targeting miRNA (miR166i-3p) was by 1.9 log fold. This converse pattern of fold change expression of all targets and their targeting miRNAs is well explained in Fig. 7. This link of miRNAs and their targets suggest the role of miRNAs in regulation of genes controlling the saponin synthesis in plant. Relative change in expression of miRNAs and their targets was not exactly same, this means that each target can be regulated by many miRNAs [39].

**Table 3:** List of miRNAs and their target genes for qRT-PCR

S.No.	Target Gene ID	Target Gene Annotation	miRNA
1	NODE_194214	Cycloartenol synthase (CAS)	miR319e.12
2	813,156	Cytochrome p450 90b1-like (CYP)	miR396e-5p.4
3	NODE_117321	Geranyl diphosphat synthase (GDPS)	miR159.12
4	713,070	4-Hydroxy-3-methylbut-2-enyl diphosphate reductase (HDR)	miR172c-5p
5	780,586	4-Hydroxy-3-methylbut-2-enyl diphosphate reductase (HDR)	miR398a-3p.5
6	NODE_215584	Hydroxymethylglutaryl-CoA synthase (HMGA)	miR167c.4
7	CL1275	Isopentenyl diphosphate isomerase (IPPI)	miR167g-5p
8	724,000	Mevalonate diphosphate decarboxylase (MVD)	miR319a.4
9	782,566	Phosphomevalonate kinase (PMK)	miR171a-3p.6
10	752,160	Squalene epoxidase (SE)	miR395h.3
11	NODE_73128	Squalene synthase (SQS)	miR166i-3p

## Discussion

miRNAs are small, non-coding, single stranded regulatory elements, subjected to deep research since last two decades. Recently, identified plant miRNAs were found to help in plant adaptation during stress conditions. These findings concluded that altered expressions of miRNAs regulate plant growth and development in several plant species subjected to abiotic stress conditions such as drought, salinity, extreme temperatures, nutrient deprivation, and heavy metals. So, miRNAs can be used further for genetic manipulations to make crop plants more stress tolerant. In the past few years, scientific endeavors have been directed towards understanding the post-transcriptional regulation of secondary metabolites involving miRNAs. Effective role of miRNAs to directly alter the plant biochemicals are already reported, understanding of this mechanism will help to further improve secondary metabolite concentration in plants. First such report was published in 2011, which disclosed the role of miR156 in regulating the amount of anthocyanin by targeting the *SPL* genes [40]. After this, a number of studies reported the role of miRNAs in regulation and biosynthesis of many secondary metabolties such as flavonoids, terpenoid and alkaloids [41]. Zhang et al. 2012 have shown in their landmark study that miRNA from food products can travel safely to mammalian gut and enter the bloodstream**.** This suggests the plant miRNAs can be used to regulate expression of target genes even for the treatment of many human diseases. Hence, miRNAs are reported to be used as bioengineering tool to alter gene expression in plants and animals [42]. Till now, many plants have been explored e.g. in rice, miR168 was found to bound human/mouse *low-density lipoprotein receptor adapter protein 1* (*LDLRAP1*) mRNA and reduce its protein level in liver, and consequently decrease LDL removal from mouse plasma [43]. In *Curcuma longa,* ath-miR167d homolog was found to hybridize with *EIF2AK2* (Tyrosine-protein kinase) and *ZFYVE16* (Zinc Finger FYVE-Type) genes responsible for blocking the pathway of protein processing in endoplasmic reticulum and regulation of membrane trafficking in the endosomal pathway. This can be helpful in treating diseases like arteriosclerosis and hyperglycemia [44]. In *Gmelina arborea* **,** 6 putative miRNAs were found to play a significant role in preventing diseases like cancer, blood borne disease, and other urinary infections [45]. Apart from this, 36 medicinal plants belonging to families like Fabaceae, Asteraceae, Brassicaceae, Theaceae, Caricaceae, Apocyanaceae, Rutaceae, Rubiaceae, Zingiberaceae, Scrophulariaceae, Myrtaceae, Verbenaceae, Linaceae, Euphorbiaceae, Solanaceae, Araliaceae, Coniferophyta, Salicaceae, Lamiaceae, Malvaceae, Ericaceae, Vitaceae, and Gramineae have been studied to explore their miRNA pool [46]. This is the first attempt to study miRNA of a medicinal monocot herb from Liliaceae family. Extensive therapeutic and medicinal properties of *C. borivilianum* made it a choice for miRNA study. *C. borivilianum* has been focused for the study of secondary metabolites biosynthetic pathways since a decade. Till now, miRNAs and their function in *C. borivilianum* are not known. Knowledge of miRNA can help in understanding the loop hole in other molecular and metabolic studies in this plant. In this study, we applied deep sequencing combined with bioinformatic analysis to identify and characterize miRNAs and their targets in *C. borivilianum*. There is no information present worldwide about sRNA of any liliaceae family plant. So this study may serve as a foundation for further exploration and application of complex metabolic mechanism of bioactive substances found in *C. borivilianum*. Larger amount of data produced by deep sequencing helped to identify even less abundant and small sized miRNAs, because analyzing larger amount of reads increases the odds of recovering rare transcripts [47].

A total of 442 known miRNAs belonging to 47 families and 5 novel miRNAs were identified from *C. borivilianum* leaf sRNA library. It signifies that, diverse and highly complex small RNA population exists in *C. borivilianum*. An almost similar trend has been observed in other monocot plants e.g. in *Aegilops tauschii*, *Brachypodium distachyon*, *O. sativa*, *Sorghum bicolor, Triticum aestivum,* and *Zea mays*, number of mature miRNAs reported are 173, 525, 713, 241, 119, and 321, respectively [27]. Although the miRNA gene sequences may vary, the seed regions of the miRNAs belonging to a same family can be identical [48]. Another difficulty regarding miRNA annotation is the repetitive presence of paralogous MIRNA loci in genome producing identical or nearly identical mature miRNAs. Based on this rationale, these miRNAs can be categorized into same families [49]. Highly conserved miRNAs like miR156, miR157, miR159, miR160, miR162, miR164, miR165, miR166, miR167, miR168, miR169, miR171, miR172, miR319, miR390, miR393, miR394, miR395, miR396, miR397, miR398, miR399, miR408, and miR444 in other species [50] were also observed in *C. borivilianum* in high abundance. Novel miRNAs or species specific miRNAs considered to play specific functions in plants whereas conserved miRNAs are thought to be involved in generalized functions like signal transduction, development of leaf and flower etc. [51]. Conserved miRNAs have unique property of being represented by multiple loci in sequenced genomes; most of them were generated through genome duplication events, giving some indication of their antiquity [52].

Conservation pattern of miRNAs vary from species to species. Most often conservation of miRNAs is associated with its sequence features such as base content and cleavage sites**.** Plant miRNAs show a negative/positive correlation between conservation and AU/GC content. At the 5′ end, conserved miRNAs usually starts with base U, while less-conserved miRNAs have a non-U base at start position in mammals. But this is not true in case of insects and plants [48]. On the basis of data analysis, it can be predicted that miR159 and miR166 have maximum expression in leaf during the period of its active growth. Highest abundance of same miRNAs is also reported in other plants like *Panax ginseng, Stevia rebaudiana,* and *A. thaliana* [53–55]. miR159 represents one of the most ancient miRNAs in the plant kingdom [52]. miR159 family members were reported to regulate ABA stress response and seed germination in plants by regulating the level of MYB transcription factor in *A. thaliana* [56, 57]. In tomato, miR159 regulates leaf and flower development by targeting the SGN-U567133 [58]. As the sample was collected during the active plant growth period, high abundance of miR159 suggests its active regulation of leaf and root development.

Monocot specific miRNAs such as miR437, miR444, miR396 were reported in monocot plant species like rice, maize, sorghum, and sugarcane [50]**.** In our study, we found the presence of only miR396 and miR444 but not miR437.

Complete reference genome should be the primary limiting factor for sRNA-seq. Unfortunately, the genome of *C. borivilianum* has not been published, but transcriptomic study of root and leaf tissue of *C. borivilianum* has already been conducted. Transcriptomic data from previous studies was used as reference for novel miRNA prediction. But many more miRNAs from *C. borivilianum* can be annotated in future when complete genome information will be available. Further, our study revealed 5 putative novel miRNAs in *C. borivilianum* using the available sequence data of this plant as reference. MFE (Minimum Fold Energy), sequence length and base composition are important features in predicting plant miRNAs. So, predicted novel miRNAs were confirmed by forming secondary structure of their precursor. All novel miRNA precursors have stem loop hairpin structure, and this fold-back hairpin structure has a low free energy as predicted by Mfold software. Novel miRNAs in *C. borivilianum* found to originate from precursor with AU percentage ranged from 40 to 53%. Seed region of miRNA can bind to the 3’-UTR, 5’-UTR, and ORF region of target mRNA but out of all these 3’-UTR targeting is much more frequent than other two [59].

In the present study, along with transcriptomic data of leaf and root of *C. borivilianum*, *O. sativa* and *A. thaliana* transcriptome have also been used to find maximum miRNAs targets. Target inhibition by cleavage was found to be more frequent. This result supports the fact that cleavage by Argonaute2 mediation maybe the main mode of gene suppression for many known plant miRNAs [60]. Moreover, we found that in *A. thaliana* ARF6 and ARF8 were targeted by miR167 and ARF10, ARF16, and ARF17 by miR160 and in *O. sativa* ARF16 by miR160; ARF6, ARF8 were targeted by miR167*.* In *C. borivilianum* ARF18 and ARF17 were found to be targeted by miR160; and ARF12 by miR167. Down-regulation of ARF6 and ARF8 by miR167 in tomato, leads to affects the floral development and female sterility [61]. This suggests the functional similarity of miRNAs in *C. borivilianum, O. sativa,* and *A. thaliana*.

Pathway specific target gene prediction in this study shown, miRNA can target transcripts coding for all-trans-nonaprenyl-diphosphate synthase, farnesyl diphosphate synthase, geranylgeranyl diphosphate synthase, hydroxymethylglutaryl-CoA synthase, isopentenyl-diphosphate delta-isomerase, 1-deoxy-D-xylulose-5-phosphate synthase, phosphomevalonate kinase, diphosphomevalonate decarboxylase, mevalonate kinase, heptaprenyl diphosphate synthase, and (E)-4-hydroxy-3-methylbut-2-enyl-diphosphate synthase.

Targets of known miRNAs from leaf transcriptome of *C. borivilianum* were found to code for protein toll, ARF, transcription factor GAMYB, growth-regulating factor, D-tyrosyl-tRNA(Tyr) deacylase, SPL protein, transport protein Sec, NAC domain-containing protein, HD-Zip protein, UDP-glucuronate decarboxylase protein, putative ABC1 protein, ethylene-insensitive 2 isoform, UDP-N-acetylglucosamine diphosphorylase, leucine-rich repeat receptor-like serine/threonine-protein kinase, tryptophan synthase, DEA(D/H)-box RNA helicase family protein, and phosphoenolpyruvate phosphatase-like. During target analysis, it was observed that the some miRNAs that were previously reported to regulate primary metabolic pathway are regulating both primary and secondary metabolic pathways in *C. borivilianum*. For example, miR172d-3p.2 target ABC1 protein and Chloroplast 1-deoxy-d-xylulose-5-phosphate synthase; miR172c target APETALA2-like protein and Farnesyl pyrophosphate synthase; miR172c-5p Ethylene-Insensitive 2 isoform X2 and 4-Hydroxy-3-methylbut-2-enyl diphosphate reductase; miR156g.2, miR156e.3 and miR156m.3 target SPL12 and Farnesyl pyrophosphate synthase. Detailed list of such miRNAs are mentioned in Additional file 9.

Target prediction results were further analyzed using Blast2GO. Biological processes like signaling, response to stimulus, developmental process, reproductive process, were found to be regulated by miRNAs. This suggested the wide regulation by miRNA in *C. borivilianum*. The two most important pathways identified were terpenoid backbone biosynthesis and sesquiterpenoid and triterpenoid biosynthesis. This suggested the specific role of miRNAs in secondary metabolism. Predicted target genes and the functions of their products may provide valuable clues for research into essential biological processes and metabolism in *C. borivilianum*.

RT-qPCR was carried out to calculate the expression levels of miRNAs and their target mRNAs. The expression was measured in leaves at two growth stages of plant. All 11 miRNAs were found with increased expression during dormancy. Maximum variation was observed in the expression of miR172c-5p and miR398a-3p.5 which are targeting 4-Hydroxy-3-methylbut-2-enyl diphosphate reductase. This concluded that multiple miRNAs can regulate same gene collectively. Respective up and down regulation of miRNAs and their targets during dormancy indicate vital role played by miRNAs in regulation of secondary metabolite accumulation. It ensures that miRNAs are directly involved in negative regulation. This present study follows the same scenario as discussed previously by some other investigators [41].

## Conclusion

Total 442 known and 5 novel miRNAs were identified with the help of bioinformatic tools. The plant specific miRNA and known miRNAs were found to be associated with MVA/MEP pathway. This helped to establish a correlation between the miRNA and secondary metabolism. miRNAs targeting genes involved in secondary metabolite biosynthetic pathway can be used to further elaborate the regulation of genes by miRNAs to moderate their expression. Our study also found some mRNA coding transcription factors as target of miRNAs which suggested the role of miRNAs in plant growth and development. GO and KEGG analysis provide foundation for further research. RT-qPCR results imply that during dormancy, synthesis of secondary metabolites slows down and helped to understand the role of miRNAs in secondary metabolism. This will facilitate to develop database of *C. borivilianum* useful for designing RNAi experiments to regulate secondary metabolite content.

## Additional files

Additional file 1: List of primer sequences for stem-loop RT-PCR of miRNAs along with miRNA sequence. (DOCX 16 kb)

Additional file 2: Known miRNAs identified in young leaf small RNA population of *C. borivilianum*. (XLSX 31 kb)

Additional file 3: Frequency percentage of each length of miRNA in *C. borivilianum. (TIFF 23 kb)*

Additional file 4: Sequencing result of stem-loop RT-PCR. (DOCX 11 kb)

Additional file 5: Identified targets of known miRNAs from leaf and root transcriptome of *C. borivilianum* using psRNATarget. (DOCX 77 kb)

Additional file 6: Identified targets of known miRNAs from *Oryza sativa* transcriptome using psRNATarget. (XLSX 88 kb)

Additional file 7: Identified targets of known miRNAs from *Arabidopsis thaliana* transcriptome using psRNATarget. (XLSX 81 kb)

Additional file 8: Saponin biosynthetic pathway specific targets from root and leaf transcriptome using miRanda. (DOCX 23 kb)

Additional file 9: List of miRNAs involved in regulating both primary and secondary metabolism. (DOCX 15 kb)

## Abbreviations

^0^C: Degree celsius
*A. thaliana*: *Arabidopsis thaliana*
ACA: 1′-acetoxychavicol acetate
ARF: Auxin respose factor
BLAST: Basic local alignment search tool
bp: Base pair
BP: Biological Process
*C. borivilianum*: *Chlorophytum borivilianum*
CC: Cellular Component
dG: Free energy
DOXP: 1-deoxy-D-xylulose 5-phosphate
EIF2AK2: Tyrosine-protein kinase
GO: Gene Ontology
Hiseq: High throughput sequencing
hsp: Length for complementarities scoring
KEGG: Kyoto encyclopedia of genes and genomes
LDLRAP1: Low-density lipoprotein receptor adapter protein 1
MEP: Non-mevalonate
MF: Molecular function
MFEs: Minimum fold energies
mg: Milli gram
MVA: Mevalonic acid
nt: Nucleotide
*O. sativa*: *Oryza sativa*
SPL: Squamosa promoter binding protein-like
TAIR: The arabidopsis information resource
UPE: Target accessibility–maximum energy to unpair the target site
ZFYVE16: Zinc finger FYVE-Type
μl: Micro liter
