# Supplementary material for: Small RNA profiling for identification of miRNAs involved in regulation of saponins biosynthesis in Chlorophytum borivilianum
Source: BMC Plant Biol. 2017 Dec 28;17:265. doi: 10.1186/s12870-017-1214-0 (PMC5745966; doi:10.1186/s12870-017-1214-0)
Supplement: Supplementary file 1 — List of primer sequences for stem-loop RT-PCR of miRNAs along with miRNA sequence. (DOCX 16 kb) [file 12870_2017_1214_MOESM1_ESM.docx]

Additional File 1: List of primer sequences for stem-loop RT-PCR of miRNAs along with miRNA sequence and qRT-PCR based expression analysis.

| miRNA | Primer sequence |
| --- | --- |
|  |  |
| miR171a-3p.1  (18bp) | miRNA: TGATTGAGCCGTGCCAAT  Forward primer: 5’-GCATGATTGAGCCGTGCCAAT-3’  RT Primer: GTCGTATCCAGTGCAGGGTCCGAGGTATTCGCACTGGATACGACA TTGGC |
| miR171a-3p.2  (19bp) | miRNA: TGATTGAGCCGTGCCAATA  Forward primer: GCATGATTGAGCCGTGCCAAT A  RT Primer: GTCGTATCCAGTGCAGGGTCCGAGGTATTCGCACTGGATACGACTATTGG |
| miR171a-3p.6  (21bp) | miRNA: TGATTGAGCCGTGCCAATATC  Forward primer: GCATGATTGAGCCGTGCCAAT A  RT Primer: GTCGTATCCAGTGCAGGGTCCGAGGTATTCGCACTGGATACGACGATATT |
| miR172c-5p | miRNA: GTGGCATCATCAAGATTCACA  Forward primer: GCAGTGGCATCATCAAGATTCAC  RT Primer: GTCGTATCCAGTGCAGGGTCCGAGGTATTCGCACTGGATACGACTGTGAA |
| miR9662a-3p | miRNA: TTGAACATCCCAGAGCCACCG  Forward primer: TTGAACATCCCAGAGCCACC  RT Primer: GTCGTATCCAGTGCAGGGTCCGAGGTATTCGCACTGGATACGACCGGTGG |
| cbo-miR1 | miRNA: AATGACTTGCGGACGTCTAGACGT  Forward primer: CACAATGACTTGCGGACGTCT  RT Primer: GTCGTATCCAGTGCAGGGTCCGAGGTATTCGCACTGGATACGACACGTCT |
| cbo-miR2 | miRNA: ATCCGCATCCGAATCCGAATCCGC  Forward primer: GCAATCCGCATCCGAATCCGA  RT Primer: GTCGTATCCAGTGCAGGGTCCGAGGTATTCGCACTGGATACGACGCGGAT |
| cbo-miR3 | miRNA: GCGGTGACGGATCTGCTTTTC  Forward primer: CACGCGGTGACGGATCTGCTT  RT Primer: GTCGTATCCAGTGCAGGGTCCGAGGTATTCGCACTGGATACGAAAAG |
| cbo-miR4 | miRNA: AAAAGCGGATTCGGATTCGGATGC  Forward primer: GCAAAAAGCGGATTCGGATTC  RT Primer: GTCGTATCCAGTGCAGGGTCCGAGGTATTCGCACTGGATACGCATCC |
| cbo-miR5 | miRNA: CGACTCCGTCGACCTTTTCTGA  Forward primer: CACGCACGACTCCGTCGACCTT  RT Primer: GTCGTATCCAGTGCAGGGTCCGAGGTATTCGCACTGGATACTCAGAA |
| Universal Reverse Primer | CCA GTG CAG GGT CCG AGG TA |
| **List of primer sequences for stem-loop qRT-PCR of miRNAs and targets.** | |
| Gene ID/miRNA | Primers |
| 752160 | Forward Primer: 5'-TGCAGCTGACTAGGAATCTGTGGAG-3' |
|  | Reverse Primer: 5'-TCGATGTTTAGTTGATGTGCCTGGG-3' |
| 782566 | Forward Primer: 5'-ACGGGAGATTAACAGCACCAAGGTA-3' |
|  | Reverse Primer: 5'-TCCACCCTTCTCGTCAATCACCTTT-3' |
| 724000 | Forward Primer: 5'-GGAGATTGAGCTAACTGGAGGGAGG-3' |
|  | Reverse Primer: 5'-CCAATCCTCCTTCCTAATCTTGACCC-3' |
| 813156 | Forward Primer: 5'-TCCTGGCAAGTTGAGAGGGATTGAA-3' |
|  | Reverse Primer: 5'-GGCCGTGAAGATCCTTGATTCTTGG-3' |
| 713070 | Forward Primer: 5'-GCTCAAAGGCGAAACAGAAGAAATAGGT-3' |
|  | Reverse Primer: 5'-CATGGCATCTTGTCTCTCCTGGGTG-3' |
| 780586 | Forward Primer: 5'-GGAATGTGACGGTGAAGCTAGCG-3' |
|  | Reverse Primer: 5'-AGTGATCCAGATTCTCTCCTCCGGA-3' |
| NODE_73128 | Forward Primer: 5'-AATGCGACGTGGTCTTACTGCTAAG-3' |
|  | Reverse Primer: 5'-AAGAGTAAGGGCAGCATTCGGATCA-3' |
| NODE_194214 | Forward Primer: 5'-GGACAGGCTGAGAGAGATCCAAAGT-3' |
|  | Reverse Primer: 5'-AACTCCCATGATTTCTTGTTGCGGG-3' |
| NODE_117321 | Forward Primer: 5'-ACCATCGAGCGTAATCTGTCTCCAA-3' |
|  | Reverse Primer: 5'-TGGGACTCAGGTTGTTTCCATGTCA-3' |
| CL1275 | Forward Primer: 5'-GCCCTTCTCAACATGGTCCCAC-3' |
|  | Reverse Primer: 5'-CTGAAGGAGCTGTTGAGGAAAGC-3' |
| NODE_215584 | Forward Primer: 5'-CACTGAAGTCGACTCCTTGTACCGA-3' |
|  | Reverse Primer: 5'-ATAAGAGACCACCGTACGTCAGTGC-3' |
| β-Actin | Forward Primer: 5'-AGCTCCTGCTCATAGTCAAG-3' |
|  | Reverse Primer: 5'-CCTATCTACGAAGGGTATGC-3' |
| miR395h.3 | RT Primer:  5'-GTCGTATCCAGTGCAGGGTCCGAGGTATTCGCACTGGATACGACGAGTTC-3' |
|  | Forward Primer: 5'-GCATGAAGTGTTTGGGGGAAC-3' |
| miR319a.4 | RT Primer:  5'-GTCGTATCCAGTGCAGGGTCCGAGGTATTCGCACTGGATACGACGGGAGC-3' |
|  | Forward Primer: 5'-GCATTGGACTGAAGGGAGCTC-3' |
| miR396e-5p.4 | RT Primer:  5'-GTCGTATCCAGTGCAGGGTCCGAGGTATTCGCACTGGATACGACAAGTTC-3' |
|  | Forward Primer: 5'-GCATTCCACAGCTTTCTTGAA-3' |
| miR172c-5p | RT Primer:  5'-GTCGTATCCAGTGCAGGGTCCGAGGTATTCGCACTGGATACGACTGTGAA-3' |
|  | Forward Primer: 5'-GCAGTGGCATCATCAAGATTC-3' |
| miR398a-3p.5 | RT Primer:  5'-GTCGTATCCAGTGCAGGGTCCGAGGTATTCGCACTGGATACGACCAGGGG-3' |
|  | Forward Primer: 5'-GCATGTGTTCTCAGGTCGCCC-3' |
| miR171a-3p.6 | RT Primer:  5'-GTCGTATCCAGTGCAGGGTCCGAGGTATTCGCACTGGATACGACGATATT-3' |
|  | Forward Primer: 5'-GCATGATTGAGCCGTGCCAAT-3' |
| miR166i-3p | RT Primer:  5'-GTCGTATCCAGTGCAGGGTCCGAGGTATTCGCACTGGATACGACGAGGAA-3' |
|  | Forward Primer: 5'-GCATCGGATCAGGCTTCATTC-3' |
| miR319e.12 | RT Primer:  5'-GTCGTATCCAGTGCAGGGTCCGAGGTATTCGCACTGGATACGACAGGAGC-3' |
|  | Forward Primer: 5'-GCATTTGGACTGAAGGGAGCT-3' |
| miR159.12 | RT Primer:  5'-GTCGTATCCAGTGCAGGGTCCGAGGTATTCGCACTGGATACGACTAGAGC-3' |
|  | Forward Primer: 5'-GCATTTGGTTTGAAGGGAGCT-3' |
| miR167g-5p | RT Primer:  5'-GTCGTATCCAGTGCAGGGTCCGAGGTATTCGCACTGGATACGACAAGATC-3' |
|  | Forward Primer: 5'-GGCGTGAAGCTGCCAGC-3' |
| miR167c.4 | RT Primer:  5'-GTCGTATCCAGTGCAGGGTCCGAGGTATTCGCACTGGATACGACAGATCA-3' |
|  | Forward Primer: 5'-GGCGTGAAGCTGCCAGC-3' |
