# Supplementary figures and images for: Small RNA profiling for identification of miRNAs involved in regulation of saponins biosynthesis in Chlorophytum borivilianum
Source: BMC Plant Biol. 2017 Dec 28;17:265. doi: 10.1186/s12870-017-1214-0 (PMC5745966; doi:10.1186/s12870-017-1214-0)

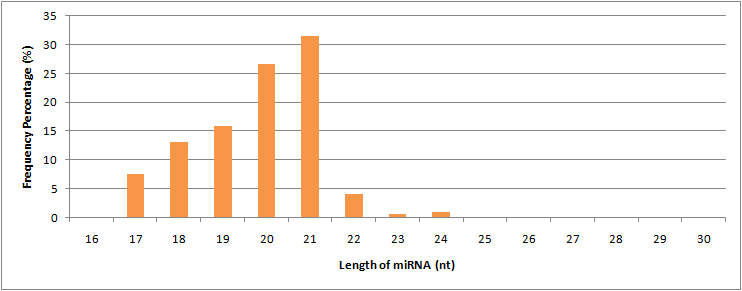

Supplement: Supplementary file 3 — Frequency percentage of each length of miRNA in C. borivilianum. (TIFF 23 kb) [file 12870_2017_1214_MOESM3_ESM.tif]
