# Supplementary material for: Small RNA profiling for identification of miRNAs involved in regulation of saponins biosynthesis in Chlorophytum borivilianum
Source: BMC Plant Biol. 2017 Dec 28;17:265. doi: 10.1186/s12870-017-1214-0 (PMC5745966; doi:10.1186/s12870-017-1214-0)
Supplement: Supplementary file 4 — Sequencing result of stem-loop RT-PCR. (DOCX 11 kb) [file 12870_2017_1214_MOESM4_ESM.docx]

Table: Sequencing result of stem-loop RT-PCR.

| miRNA | Sequence |
| --- | --- |
| miRNA172c | GCAGTGGCATCATCAAGATTCACAGTCGTATCCAGTGCGAATACCTCGGACCCTGCACTGGATACGAC |
| miR171a-3p.1  (18bp) | GCATGATTGAGCCGTGCCAATGTCGTATCCAGTGCGAATACCTCGGACCCTGCTGGATACGACA |
| miR171a-3p.2  (19bp) | GCATGATTGAGCCGTGCCAATAGTCGTATCCAGTGCGAATACCTCGGACCCTGCACTGGATACGACA |
| miR171a-3p.6  (21bp) | GCATGATTGAGCCGTGCCAATACCTCGGACCCTGCACTGG |
| miRNA9662a-3p | TTGAACATCCCAGAGCCACCGGTCGTATCCAGTGCGAATACCTCGGACCCTGCACTGGATACGACA |
| cbo-miR1 | CCAGTGCAGGGTCCGAGGTATTCGCACTGGATACGAGACGTCCGCAAGTCATTGTGA |
| cbo-miR2 | ATCCGCATCCGAATCCGAATCCGCGTCGTATCCAGTGCGAATACCTCGGACCCTGCACTGGATACGACA |
| cbo-miR3 | CACGCGGTGACGGATCTGCTTTTCGTCGTATCCAGTGCGAATACCTCGGACCCTGCACTGGATACGAC |
| cbo-miR4 | GTCGTATCCAGTGCAGGGTCCGAGGTATTCGCACTGGATACGCATCCGAATCCGAATCCGCTTTTTGCA |
| cbo-miR5 | CACGACTCCGTCGACCTTTTCTGAGTCGTATCCAGTGCGAATACCTCGGACCCTGCACTGGATACGAC |
