# Supplementary material for: Small RNA profiling for identification of miRNAs involved in regulation of saponins biosynthesis in Chlorophytum borivilianum
Source: BMC Plant Biol. 2017 Dec 28;17:265. doi: 10.1186/s12870-017-1214-0 (PMC5745966; doi:10.1186/s12870-017-1214-0)
Supplement: Supplementary file 5 — Identified targets of known miRNAs from leaf and root transcriptome of C. borivilianum using psRNATarget. (DOCX 77 kb) [file 12870_2017_1214_MOESM5_ESM.docx]

Table: Identified targets of known miRNAs from leaf and root transcriptome of *C. borivilianum* using psRNATarget.

| Target description | Target ID | miRNA | Expectation | UPE | Inhibition |
| --- | --- | --- | --- | --- | --- |
| APETALA2-like protein | NODE_74849_length_1243_cov_2.761867 | miR172a-3p.2 | 1.5 | -1 | Cleavage |
|  |  | miR172a-3p.1 | 1.5 | -1 | Cleavage |
|  |  | miR172c | 1.5 | -1 | Cleavage |
|  |  | miR172d-3p.2 | 1.5 | -1 | Cleavage |
|  |  | miR172d.2 | 1.5 | -1 | Cleavage |
|  |  | miR172d.4 | 1.5 | -1 | Cleavage |
|  |  | miR172d.3 | 1.5 | -1 | Cleavage |
|  |  | miR172d.5 | 0 | -1 | Cleavage |
|  |  | miR172d.6 | 1.5 | -1 | Cleavage |
|  | NODE_29665_length_435_cov_2.455172 | miR172a-3p.2 | 2.5 | -1 | Cleavage |
|  |  | miR172a-3p.1 | 2.5 | -1 | Cleavage |
|  |  | miR172c | 2.5 | -1 | Cleavage |
|  |  | miR172d.2 | 2.5 | -1 | Cleavage |
|  |  | miR172d.4 | 2.5 | -1 | Cleavage |
|  |  | miR172d.3 | 2.5 | -1 | Cleavage |
|  |  | miR172d.5 | 1 | -1 | Cleavage |
| Auxin response factor 12-like | NODE_27675_length_719_cov_2.810848 | miR167.1 | 2.5 | -1 | Cleavage |
|  |  | miR167c.9 | 2.5 | -1 | Cleavage |
|  |  | miR167c.5 | 2.5 | -1 | Cleavage |
|  |  | miR167c.7 | 2.5 | -1 | Cleavage |
|  |  | miR167c.6 | 2.5 | -1 | Cleavage |
|  |  | miR167f-5p.1 | 2.5 | -1 | Cleavage |
|  |  | miR167g.1 | 1.5 | -1 | Cleavage |
| Auxin response factor 17 isoform X2 | 808642 | miR167.1 | 2.5 | -1 | Cleavage |
|  |  | miR167c.9 | 2.5 | -1 | Cleavage |
|  |  | miR167c.5 | 2.5 | -1 | Cleavage |
|  |  | miR167c.7 | 2.5 | -1 | Cleavage |
|  |  | miR167c.6 | 2.5 | -1 | Cleavage |
|  |  | miR167f-5p.1 | 2.5 | -1 | Cleavage |
|  |  | miR167g.2 | 2.5 | -1 | Cleavage |
|  |  | miR167g.1 | 2.5 | -1 | Cleavage |
| Auxin response factor 18-like isoform X2 [Asparagus officinalis] | NODE_185800_length_896_cov_2.824777 | miR160b-5p.3 | 0 | -1 | Cleavage |
|  |  | miR160b-5p.2 | 0 | -1 | Cleavage |
|  |  | miR160b-5p.3 | 0 | -1 | Cleavage |
|  |  | miR160b-5p.4 | 0 | -1 | Cleavage |
|  |  | miR160e-5p | 0 | -1 | Cleavage |
|  |  | miR160h | 0 | -1 | Cleavage |
| Auxin response factor 18 | 689634 | miR160b-5p.3 | 0 | -1 | Cleavage |
|  |  | miR160b-5p.2 | 0 | -1 | Cleavage |
|  |  | miR160b-5p.4 | 0 | -1 | Cleavage |
|  |  | miR160e-5p | 0 | -1 | Cleavage |
|  |  | miR160h | 0 | -1 | Cleavage |
| auxin response factor 18-like isoform X2 | 793746 | miR160b-5p.3 | 0 | -1 | Cleavage |
|  |  | miR160b-5p.2 | 0 | -1 | Cleavage |
|  |  | miR160b-5p.4 | 0 | -1 | Cleavage |
|  |  | miR160e-5p | 0 | -1 | Cleavage |
|  |  | miR160h | 0 | -1 | Cleavage |
|  |  |  |  |  |  |
| Low affinity sulfate transporter 3 | NODE_55475_length_1771_cov_2.926030 | miR395b.1 | 0 | -1 | Cleavage |
|  |  | miR395b.2 | 2 | -1 | Cleavage |
|  |  | miR395b.3 | 1 | -1 | Cleavage |
|  |  | miR395e.2 | 0 | -1 | Cleavage |
|  |  | miR395e.1 | 0 | -1 | Cleavage |
|  |  | miR395h.2 | 0 | -1 | Cleavage |
|  |  | miR395h.3 | 0 | -1 | Cleavage |
|  |  | miR395h.4 | 1 | -1 | Cleavage |
|  |  | miR395i.3 | 1 | -1 | Cleavage |
|  |  | miR395i.4 | 1 | -1 | Cleavage |
|  |  | miR395i.2 | 1 | -1 | Cleavage |
|  |  | miR395k | 1.5 | -1 | Cleavage |
| MADS-box transcription factor 23-like isoform X3 | NODE_22586_length_655_cov_2.364886 | miR444b.1 | 0 | -1 | Cleavage |
|  | NODE_187813_length_439_cov_8.000000 | miR444b.1 | 1 | -1 | Cleavage |
|  | NODE_60565_length_442_cov_2.432127 | miR444b.1 | 1.5 | -1 | Cleavage |
| Signal transduction histidine kinase | NODE_52348_length_181_cov_3.176795 | miR473 | 0 | -1 | Cleavage |
|  |  | miR477a.1 | 0 | -1 | Cleavage |
|  |  | miR477a.2 | 0 | -1 | Cleavage |
|  |  | miR477e | 0 | -1 | Cleavage |
|  |  | miR477h.3 | 0 | -1 | Cleavage |
|  |  | miR477h.4 | 0 | -1 | Cleavage |
|  |  | miR477i | 0 | -1 | Cleavage |
| squamosa promoter-binding-like protein 12 | NODE_164040_length_1637_cov_5.246793 | miR156 | 2 | -1 | Cleavage |
|  |  | miR156a.1 | 2 | -1 | Cleavage |
|  |  | miR156b | 1 | -1 | Cleavage |
|  |  | miR156c.1 | 2.5 | -1 | Cleavage |
|  |  | miR156c.2 | 1 | -1 | Cleavage |
|  |  | miR156e.3 | 1 | -1 | Cleavage |
|  |  | miR156e-5p | 2.5 | -1 | Cleavage |
|  |  | miR156e.2 | 2.5 | -1 | Cleavage |
|  |  | miR156f.4 | 1.5 | -1 | Cleavage |
|  |  | miR156f.5 | 1.5 | -1 | Cleavage |
|  |  | miR156f.3 | 1.5 | -1 | Cleavage |
|  |  | miR156f-5p | 2 | -1 | Cleavage |
|  |  | miR156g.2 | 1 | -1 | Cleavage |
|  |  | miR156j.2 | 2.5 | -1 | Cleavage |
|  |  | miR156j.3 | 1 | -1 | Cleavage |
|  |  | miR156m.3 | 1 | -1 | Cleavage |
|  |  | miR156m.2 | 1 | -1 | Cleavage |
|  |  | miR156p | 1.5 | -1 | Translation |
|  |  | miR156s | 1 | -1 | Cleavage |
| squamosa promoter-binding-like protein 7 | NODE_125089_length_716_cov_4.988827 | miR156 | 2 | -1 | Cleavage |
|  |  | miR156a.1 | 2 | -1 | Cleavage |
|  |  | miR156b | 1 | -1 | Cleavage |
|  |  | miR156c.1 | 2.5 | -1 | Cleavage |
|  |  | miR156c.2 | 1 | -1 | Cleavage |
|  |  | miR156e-5p | 2.5 | -1 | Cleavage |
|  |  | miR156e.2 | 2.5 | -1 | Cleavage |
|  |  | miR156e.3 | 1 | -1 | Cleavage |
|  |  | miR156f.4 | 2.5 | -1 | Cleavage |
|  |  | miR156f-5p | 2 | -1 | Cleavage |
|  |  | miR156f.5 | 2.5 | -1 | Cleavage |
|  |  | miR156f.3 | 2.5 | -1 | Cleavage |
|  |  | miR156g.2 | 1 | -1 | Cleavage |
|  |  | miR156j.3 | 2 | -1 | Cleavage |
|  |  | miR156j.2 | 2.5 | -1 | Cleavage |
|  |  | miR156m.3 | 1 | -1 | Cleavage |
|  |  | miR156m.2 | 1 | -1 | Cleavage |
|  |  | miR156p | 1.5 | -1 | Translation |
|  |  | miR156s | 1 | -1 | Cleavage |
| Squamosa promoter-binding-like protein 16 | 794196 | miR156b | 1 | -1 | Cleavage |
|  |  | miR156c.2 | 1 | -1 | Cleavage |
|  |  | miR156e.3 | 1 | -1 | Cleavage |
|  |  | miR156g.2 | 1 | -1 | Cleavage |
|  |  | miR156m.3 | 1 | -1 | Cleavage |
|  |  | miR156m.2 | 1 | -1 | Cleavage |
|  |  | miR156s | 1 | -1 | Cleavage |
|  |  | miR156p | 1.5 | -1 | Translation |
|  |  | miR156 | 2 | -1 | Cleavage |
|  |  | miR156a.1 | 2 | -1 | Cleavage |
|  |  | miR156f-5p | 2 | -1 | Cleavage |
|  |  | miR156j.3 | 2 | -1 | Cleavage |
|  |  | miR156c.1 | 2.5 | -1 | Cleavage |
|  |  | miR156e-5p | 2.5 | -1 | Cleavage |
|  |  | miR156e.2 | 2.5 | -1 | Cleavage |
|  |  | miR156f.4 | 2.5 | -1 | Cleavage |
|  |  | miR156f.5 | 2.5 | -1 | Cleavage |
|  |  | miR156f.3 | 2.5 | -1 | Cleavage |
|  |  | miR156j.2 | 2.5 | -1 | Cleavage |
| NAC domain-containing protein 21/22-like | NODE_62669_length_860_cov_2.422093 | miR164a.1 | 1 | -1 | Cleavage |
|  |  | miR164b.2 | 1 | -1 | Cleavage |
|  |  | miR164b.3 | 1 | -1 | Cleavage |
|  |  | miR164b.4 | 1 | -1 | Cleavage |
|  |  | miR164b.5 | 1 | -1 | Cleavage |
|  |  | miR164c | 2 | -1 | Cleavage |
|  |  | miR164c-5p | 1 | -1 | Cleavage |
|  |  | miR164h-5p.1 | 1 | -1 | Cleavage |
|  |  | miR164h-5p.2 | 1 | -1 | Cleavage |
| NAC domain-containing protein 21/22-like | 677859 | miR164a.1 | 1 | -1 | Cleavage |
|  |  | miR164a.2 | 1 | -1 | Cleavage |
|  |  | miR164b.2 | 1 | -1 | Cleavage |
|  |  | miR164b.3 | 1 | -1 | Cleavage |
|  |  | miR164b.4 | 1 | -1 | Cleavage |
|  |  | miR164b.5 | 1 | -1 | Cleavage |
|  |  | miR164c-5p | 1 | -1 | Cleavage |
|  |  | miR164e-5p | 1 | -1 | Cleavage |
|  |  | miR164h-5p.1 | 1 | -1 | Cleavage |
|  |  | miR164h-5p.2 | 1 | -1 | Cleavage |
|  |  | miR164c | 2 | -1 | Cleavage |
| HD-Zip protein ATHB-15 | NODE_38641_length_264_cov_5.946970 | miR166.1 | 2 | -1 | Cleavage |
|  |  | miR166.2 | 2 | -1 | Cleavage |
|  |  | miR165a-3p | 1 | -1 | Cleavage |
|  |  | miR165b-3p.3 | 1 | -1 | Cleavage |
|  |  | miR165b-3p.4 | 1 | -1 | Cleavage |
|  |  | miR166b.2 | 2 | -1 | Cleavage |
|  |  | miR166b.5 | 2 | -1 | Cleavage |
|  |  | miR166b.3 | 2 | -1 | Cleavage |
|  |  | miR166b.4 | 2 | -1 | Cleavage |
|  |  | miR166b.1 | 3 | -1 | Cleavage |
|  |  | miR166d.4 | 2 | -1 | Cleavage |
|  |  | miR166d.3 | 2 | -1 | Cleavage |
|  |  | miR166d.6 | 2 | -1 | Cleavage |
|  |  | miR166d.5 | 2 | -1 | Cleavage |
|  |  | miR166g-3p.1 | 2 | -1 | Cleavage |
|  |  | miR166g-3p.2 | 2 | -1 | Cleavage |
|  |  | miR166g-3p.3 | 2 | -1 | Cleavage |
|  |  | miR166h.1 | 2 | -1 | Cleavage |
|  |  | miR166i.1 | 3 | -1 | Cleavage |
|  |  | miR166i.3 | 3 | -1 | Cleavage |
|  |  | miR166l.2 | 1 | -1 | Cleavage |
|  |  | miR166l.1 | 1 | -1 | Cleavage |
|  |  | miR166o | 2 | -1 | Cleavage |
| HD-Zip protein ATHB-15 | 714734 | miR166.2 | 2 | -1 | Cleavage |
|  |  | miR166b.5 | 2 | -1 | Cleavage |
|  |  | miR166b.3 | 2 | -1 | Cleavage |
|  |  | miR166b.4 | 2 | -1 | Cleavage |
|  |  | miR166d.3 | 2 | -1 | Cleavage |
|  |  | miR166d.6 | 2 | -1 | Cleavage |
|  |  | miR166d.5 | 2 | -1 | Cleavage |
|  |  | miR166g-3p.1 | 2 | -1 | Cleavage |
|  |  | miR166g-3p.3 | 2 | -1 | Cleavage |
|  |  | miR166h.1 | 2 | -1 | Cleavage |
|  |  | miR166h.2 | 2 | -1 | Cleavage |
|  |  | miR166o | 2 | -1 | Cleavage |
|  |  | miR166i.1 | 3 | -1 | Cleavage |
|  |  | miR166i.3 | 3 | -1 | Cleavage |
|  |  | miR165a-3p | 1 | -1 | Cleavage |
|  |  | miR165b-3p.3 | 1 | -1 | Cleavage |
|  |  | miR165b-3p.4 | 1 | -1 | Cleavage |
|  |  | miR166l.2 | 1 | -1 | Cleavage |
|  |  | miR166l.1 | 1 | -1 | Cleavage |
| Class III homeobox-leucine zipper protein | NODE_22647_length_2688_cov_5.982887 | miR166.1 | 2 | -1 | Cleavage |
|  |  | miR166.2 | 2 | -1 | Cleavage |
|  |  | miR165a-3p | 1 | -1 | Cleavage |
|  |  | miR166b.1 | 3 | -1 | Cleavage |
|  |  | miR165b-3p.3 | 1 | -1 | Cleavage |
|  |  | miR165b-3p.4 | 1 | -1 | Cleavage |
|  |  | miR166b.2 | 2 | -1 | Cleavage |
|  |  | miR166b.5 | 2 | -1 | Cleavage |
|  |  | miR166b.3 | 2 | -1 | Cleavage |
|  |  | miR166b.4 | 2 | -1 | Cleavage |
|  |  | miR166d.4 | 2 | -1 | Cleavage |
|  |  | miR166d.3 | 2 | -1 | Cleavage |
|  |  | miR166d.6 | 2 | -1 | Cleavage |
|  |  | miR166d.5 | 2 | -1 | Cleavage |
|  |  | miR166g-3p.1 | 2 | -1 | Cleavage |
|  |  | miR166g-3p.2 | 2 | -1 | Cleavage |
|  |  | miR166g-3p.3 | 2 | -1 | Cleavage |
|  |  | miR166i.1 | 3 | -1 | Cleavage |
|  |  | miR166i.3 | 3 | -1 | Cleavage |
|  |  | miR166l.2 | 1 | -1 | Cleavage |
|  |  | miR166l.1 | 1 | -1 | Cleavage |
|  |  | miR166o | 2 | -1 | Cleavage |
| Scarecrow-like protein 6 | NODE_160503_length_218_cov_5.899083 | miR171a-3p.3 | 2.5 | -1 | Cleavage |
|  |  | miR171a | 2.5 | -1 | Translation |
|  |  | miR171b-3p.2 | 2.5 | -1 | Translation |
|  |  | miR171b-3p.1 | 2.5 | -1 | Translation |
|  |  | miR171c-3p.1 | 2.5 | -1 | Cleavage |
|  |  | miR171j | 2.5 | -1 | Cleavage |
|  |  | miR171m | 2.5 | -1 | Translation |
|  |  | miR171o-3p | 2.5 | -1 | Cleavage |
|  |  | miR171b | 1 | -1 | Cleavage |
|  |  | miR171a-3p.2 | 1.5 | -1 | Cleavage |
|  |  | miR171a-3p.6 | 1.5 | -1 | Cleavage |
|  |  | miR171c | 1.5 | -1 | Cleavage |
| Transport inhibitor response 1-like protein | NODE_151253_length_152_cov_4.000000 | miR393b-5p | 1 | -1 | Cleavage |
|  | NODE_151252_length_1164_cov_3.000000 | miR393b-5p | 1 | -1 | Cleavage |
|  | 763354 | miR393b-5p | 1 | -1 | Cleavage |
|  | 816548 | miR393b-5p | 1 | -1 | Cleavage |
| F-box only protein 6-like | NODE_139420_length_1125_cov_3.000000 | miR394a-5p | 1 | -1 | Cleavage |
|  |  | miR394c | 1 | -1 | Cleavage |
| SPX domain-containing membrane protein | NODE_23472_length_475_cov_2.267368 | miR827-3p | 1 | -1 | Cleavage |
|  |  | miR827 | 1 | -1 | Cleavage |
|  |  | miR827b.2 | 1 | -1 | Cleavage |
|  |  | miR827b.4 | 1 | -1 | Cleavage |
|  |  | miR827b.3 | 1 | -1 | Cleavage |
|  | 816472 | miR827-3p | 1 | -1 | Cleavage |
|  |  | miR827 | 1 | -1 | Cleavage |
|  |  | miR827b.2 | 1 | -1 | Cleavage |
|  |  | miR827b.4 | 1 | -1 | Cleavage |
|  |  | miR827b.3 | 1 | -1 | Cleavage |
| laccase-22-like | NODE_61474_length_713_cov_2.949509 | miR397 | 1.5 | -1 | Cleavage |
|  |  | miR397a.1 | 1.5 | -1 | Cleavage |
|  |  | miR397a.2 | 1.5 | -1 | Cleavage |
|  |  |  |  |  |  |
| UDP-glucuronic acid decarboxylase 6-like | NODE_172165_length_279_cov_8.000000 | miR164a.1 | 2 | -1 | Cleavage |
|  |  | miR164a.2 | 2 | -1 | Cleavage |
|  |  | miR164b.2 | 2 | -1 | Cleavage |
|  |  | miR164b.3 | 2 | -1 | Cleavage |
|  |  | miR164b.4 | 2 | -1 | Cleavage |
|  |  | miR164b.5 | 2 | -1 | Cleavage |
|  |  | miR164c-5p | 2 | -1 | Cleavage |
|  |  | miR164c | 3 | -1 | Cleavage |
|  |  | miR164e-5p | 2 | -1 | Cleavage |
|  |  | miR164h-5p.1 | 2 | -1 | Cleavage |
|  |  | miR164h-5p.2 | 2 | -1 | Cleavage |
| UDP-glucuronate decarboxylase protein 6 | 745120 | miR164a.1 | 2 | -1 | Cleavage |
|  |  | miR164a.2 | 2 | -1 | Cleavage |
|  |  | miR164b.2 | 2 | -1 | Cleavage |
|  |  | miR164b.3 | 2 | -1 | Cleavage |
|  |  | miR164b.4 | 2 | -1 | Cleavage |
|  |  | miR164b.5 | 2 | -1 | Cleavage |
|  |  | miR164c-5p | 2 | -1 | Cleavage |
|  |  | miR164e-5p | 2 | -1 | Cleavage |
|  |  | miR164h-5p.1 | 2 | -1 | Cleavage |
|  |  | miR164h-5p.2 | 2 | -1 | Cleavage |
|  |  | miR164c | 3 | -1 | Cleavage |
| UDP-N-acetylglucosamine diphosphorylase | 805902 | miR319c-3p.1 | 3 | -1 | Cleavage |
|  |  | miR319c-3p.2 | 3 | -1 | Cleavage |
|  |  | miR319e.10 | 3 | -1 | Cleavage |
|  |  | miR319e.9 | 3 | -1 | Cleavage |
|  |  | miR319l.3 | 3 | -1 | Cleavage |
|  |  | miR319l.4 | 3 | -1 | Cleavage |
|  |  | miR319e.7 | 2 | -1 | Cleavage |
|  |  | miR319l.2 | 2 | -1 | Cleavage |
|  |  | miR319l.1 | 2 | -1 | Cleavage |
|  |  | miR319a.4 | 3 | -1 | Cleavage |
|  |  | miR319a.5 | 3 | -1 | Cleavage |
|  |  | miR319a.3 | 3 | -1 | Cleavage |
|  |  | miR319a.6 | 3 | -1 | Cleavage |
| xyloglucan endotransglucosylase/hydrolase protein 24-like | NODE_81591_length_967_cov_3.008273 | miR167.2 | 2 | -1 | Cleavage |
|  |  | miR167.1 | 2 | -1 | Cleavage |
|  |  | miR167b | 2 | -1 | Cleavage |
|  |  | miR167c.7 | 3 | -1 | Cleavage |
|  |  | miR167c.3 | 2 | -1 | Cleavage |
|  |  | miR167c.4 | 2 | -1 | Cleavage |
|  |  | miR167c.8 | 2 | -1 | Cleavage |
|  |  | miR167c.11 | 2 | -1 | Cleavage |
|  |  | miR167c.9 | 2 | -1 | Cleavage |
|  |  | miR167c.5 | 2 | -1 | Cleavage |
|  |  | miR167c.6 | 2 | -1 | Cleavage |
|  |  | miR167c.10 | 2 | -1 | Cleavage |
|  |  | miR167d | 2 | -1 | Cleavage |
|  |  | miR167f-5p.2 | 2 | -1 | Cleavage |
|  |  | miR167f-5p.1 | 2 | -1 | Cleavage |
|  |  | miR167g-5p | 2 | -1 | Cleavage |
|  |  | miR167g.2 | 2 | -1 | Cleavage |
|  |  | miR167g.1 | 2 | -1 | Cleavage |
|  |  | miR167g.3 | 2 | -1 | Cleavage |
| ABC1 protein | NODE_209522_length_395_cov_2.800000 | miR172a-3p.2 | 2 | -1 | Cleavage |
|  |  | miR172a-3p.1 | 2 | -1 | Cleavage |
|  |  | miR172d.2 | 2 | -1 | Cleavage |
|  |  | miR172d.4 | 2 | -1 | Cleavage |
|  |  | miR172d.3 | 2 | -1 | Cleavage |
|  |  | miR172d-3p.2 | 3 | -1 | Cleavage |
|  |  | miR172d.6 | 3 | -1 | Cleavage |
|  | 801096 | miR172d-3p.2 | 3 | -1 | Cleavage |
|  |  | miR172d.6 | 3 | -1 | Cleavage |
|  |  | miR172a-3p.2 | 2 | -1 | Cleavage |
|  |  | miR172a-3p.1 | 2 | -1 | Cleavage |
|  |  | miR172d.2 | 2 | -1 | Cleavage |
|  |  | miR172d.4 | 2 | -1 | Cleavage |
|  |  | miR172d.3 | 2 | -1 | Cleavage |
| 5-methyltetrahydropteroyltriglutamate--homocysteine methyltransferase 2-like | NODE_1456_length_102_cov_3.000000 | miR156b-3p | 2.5 | -1 | Cleavage |
| DNA-directed RNA polymerase II subunit | NODE_194453_length_160_cov_2.937500 | miR166a-5p.4 | 2.5 | -1 | Translation |
|  |  | miR166a-5p.5 | 2.5 | -1 | Translation |
| protein argonaute 1A-like | NODE_147176_length_751_cov_8.000000 | miR168b-5p.4 | 2.5 | -1 | Cleavage |
|  |  | miR168b-5p.3 | 2.5 | -1 | Cleavage |
| phosphoenolpyruvate phosphatase-like | NODE_106248_length_778_cov_5.000000 | miR3630-3p.8 | 2.5 | -1 | Cleavage |
|  |  | miR3630-3p.6 | 2.5 | -1 | Cleavage |
|  |  | miR3630-3p.9 | 2.5 | -1 | Cleavage |
|  | 811858 | miR3630-3p.8 | 2.5 | -1 | Cleavage |
|  |  | miR3630-3p.6 | 2.5 | -1 | Cleavage |
|  |  | miR3630-3p.9 | 2.5 | -1 | Cleavage |
| choline-phosphate cytidylyltransferase 2-like | NODE_168241_length_183_cov_3.978142 | miR3630-3p.6 | 2.5 | -1 | Cleavage |
|  |  | miR3630-3p.9 | 2.5 | -1 | Cleavage |
| Mucin-19 | NODE_47834_length_213_cov_4.685446 | miR396d.2 | 2.5 | -1 | Translation |
|  |  | miR396d.6 | 2.5 | -1 | Translation |
|  |  | miR396d.3 | 2.5 | -1 | Translation |
| Mucin-19 | 768366 | miR396d.2 | 2.5 | -1 | Translation |
|  |  | miR396d.6 | 2.5 | -1 | Translation |
|  |  | miR396d.3 | 2.5 | -1 | Translation |
| DNA repair protein | NODE_132259_length_322_cov_5.000000 | miR396g-3p | 2.5 | -1 | Cleavage |
|  | 786258 | miR396a-3p.3 | 3 | -1 | Cleavage |
| Protein HUA2-LIKE 1 Enhancer | NODE_45999_length_993_cov_2.656596 | miR396g-3p | 2.5 | -1 | Cleavage |
|  | 810172 | miR396g-3p | 2.5 | -1 | Cleavage |
| Rhomboid protease glpG | NODE_177339_length_280_cov_1.817857 | miR397 | 2.5 | -1 | Cleavage |
|  |  | miR397a.1 | 2.5 | -1 | Cleavage |
|  |  | miR397a.2 | 2.5 | -1 | Cleavage |
| Altronate hydrolase | NODE_188170_length_472_cov_5.000000 | miR5205b.2 | 2.5 | -1 | Cleavage |
|  |  | miR5205b.1 | 2.5 | -1 | Cleavage |
|  |  | miR5205c.1 | 2.5 | -1 | Cleavage |
|  |  | miR5205c.2 | 2.5 | -1 | Cleavage |
| L-type lectin-domain containing receptor kinase | NODE_48445_length_202_cov_3.000000 | miR156e.2 | 3 | -1 | Cleavage |
| synaptotagmin-2-like | NODE_21955_length_1905_cov_3.966404 | miR159.10 | 3 | -1 | Cleavage |
|  |  | miR159b.10 | 3 | -1 | Cleavage |
|  |  | miR159b.7 | 3 | -1 | Cleavage |
|  |  | miR159b.5 | 3 | -1 | Cleavage |
|  |  | miR159b.9 | 3 | -1 | Cleavage |
|  |  | miR159c.5 | 3 | -1 | Cleavage |
|  |  | miR159c.7 | 3 | -1 | Cleavage |
| TRIGALACTOSYLDIACYLGLYCEROL 3, chloroplastic | NODE_156100_length_765_cov_7.000000 | miR159.9 | 3 | -1 | Cleavage |
|  |  | miR159.12 | 3 | -1 | Cleavage |
|  | 813656 | miR159.9 | 3 | -1 | Cleavage |
|  |  | miR159.12 | 3 | -1 | Cleavage |
| cysteine synthase-like | NODE_176629_length_1129_cov_6.000000 | miR166g-3p.2 | 3 | -1 | Cleavage |
|  | 811812 | miR166g-3p.2 | 3 | -1 | Cleavage |
| protein RRC1-like isoform X1 | NODE_46656_length_853_cov_2.885111 | miR169 | 3 | -1 | Cleavage |
|  |  | miR169a | 3 | -1 | Cleavage |
| TCP transcription factor 24 | NODE_66202_length_125_cov_4.576000 | miR319 | 3 | -1 | Cleavage |
|  |  | miR319e.10 | 3 | -1 | Cleavage |
|  |  | miR319e.9 | 3 | -1 | Cleavage |
|  |  | miR319l.3 | 3 | -1 | Cleavage |
|  | NODE_203145_length_277_cov_3.205776 | miR319l.3 | 3 | -1 | Cleavage |
|  |  | miR319 | 3 | -1 | Cleavage |
|  |  | miR319e.10 | 3 | -1 | Cleavage |
|  |  | miR319e.9 | 3 | -1 | Cleavage |
|  | 778442 | miR319 | 3 | -1 | Cleavage |
|  |  | miR319e.10 | 3 | -1 | Cleavage |
|  |  | miR319e.9 | 3 | -1 | Cleavage |
|  |  | miR319l.3 | 3 | -1 | Cleavage |
| Sugar transporter ERD6-like 16 | NODE_41487_length_1214_cov_3.457990 | miR394a-5p | 3 | -1 | Cleavage |
|  |  | miR394c | 3 | -1 | Cleavage |
| Mitogen-activated protein kinase 4 | NODE_219917_length_298_cov_2.741611 | miR477e | 3 | -1 | Cleavage |
| Protein toll | 757076 | miR159.10 | 0 | -1 | Cleavage |
|  |  | miR159b.10 | 0 | -1 | Cleavage |
|  |  | miR159b.7 | 0 | -1 | Cleavage |
|  |  | miR159b.5 | 0 | -1 | Cleavage |
|  |  | miR159b.9 | 0 | -1 | Cleavage |
|  |  | miR159c.8 | 0 | -1 | Cleavage |
|  |  | miR159c.5 | 0 | -1 | Cleavage |
|  |  | miR159c.7 | 0 | -1 | Cleavage |
|  |  | miR159a.10 | 1 | -1 | Cleavage |
|  |  | miR159a.7 | 1 | -1 | Cleavage |
|  |  | miR159c.4 | 1 | -1 | Cleavage |
|  |  | miR159c.3 | 1 | -1 | Cleavage |
|  |  | miR159d-3p.3 | 1 | -1 | Cleavage |
|  |  | miR159f.1 | 1 | -1 | Cleavage |
|  |  | miR159f.2 | 1 | -1 | Cleavage |
|  |  | miR159.11 | 1.5 | -1 | Cleavage |
|  |  | miR159.7 | 1.5 | -1 | Cleavage |
|  |  | miR159.9 | 1.5 | -1 | Cleavage |
|  |  | miR159.12 | 1.5 | -1 | Cleavage |
|  |  | miR159e.1 | 1.5 | -1 | Cleavage |
|  |  | miR159e.2 | 1.5 | -1 | Cleavage |
|  |  | miR159e.3 | 1.5 | -1 | Cleavage |
|  |  | miR159i-3p.2 | 1.5 | -1 | Cleavage |
|  |  | miR159i-3p.1 | 1.5 | -1 | Cleavage |
|  |  | miR319e.12 | 1.5 | -1 | Cleavage |
|  |  | miR319e.8 | 1.5 | -1 | Cleavage |
|  |  | miR319e.11 | 1.5 | -1 | Cleavage |
|  |  | miR159g-3p | 2.5 | -1 | Cleavage |
|  |  | miR319 | 2.5 | -1 | Cleavage |
|  |  | miR319c.5 | 2.5 | -1 | Cleavage |
|  |  | miR319c.3 | 2.5 | -1 | Cleavage |
|  |  | miR319c.4 | 2.5 | -1 | Cleavage |
|  |  | miR319h | 2.5 | -1 | Cleavage |
| Transcription factor GAMYB isoform X1 | 696836 | miR159i-3p.2 | 0 | -1 | Cleavage |
|  |  | miR159i-3p.1 | 0 | -1 | Cleavage |
|  |  | miR159g-3p | 1 | -1 | Cleavage |
|  |  | miR159.10 | 1.5 | -1 | Cleavage |
|  |  | miR159.11 | 1.5 | -1 | Cleavage |
|  |  | miR159.7 | 1.5 | -1 | Cleavage |
|  |  | miR159a.8 | 1.5 | -1 | Cleavage |
|  |  | miR159a.9 | 1.5 | -1 | Cleavage |
|  |  | miR159b.10 | 1.5 | -1 | Cleavage |
|  |  | miR159b.7 | 1.5 | -1 | Cleavage |
|  |  | miR159b.5 | 1.5 | -1 | Cleavage |
|  |  | miR159b.9 | 1.5 | -1 | Cleavage |
|  |  | miR159c.8 | 1.5 | -1 | Cleavage |
|  |  | miR159c.5 | 1.5 | -1 | Cleavage |
|  |  | miR159c.7 | 1.5 | -1 | Cleavage |
|  |  | miR159d-3p.2 | 1.5 | -1 | Cleavage |
|  |  | miR319a.4 | 1.5 | -1 | Cleavage |
|  |  | miR319a.6 | 1.5 | -1 | Cleavage |
|  |  | miR319c-3p.2 | 1.5 | -1 | Cleavage |
|  |  | miR319e.12 | 1.5 | -1 | Cleavage |
|  |  | miR319e.10 | 1.5 | -1 | Cleavage |
|  |  | miR319e.8 | 1.5 | -1 | Cleavage |
|  |  | miR319e.9 | 1.5 | -1 | Cleavage |
|  |  | miR319e.11 | 1.5 | -1 | Cleavage |
|  |  | miR319l.3 | 1.5 | -1 | Cleavage |
|  |  | miR319l.4 | 1.5 | -1 | Cleavage |
|  |  | miR159.6 | 2.5 | -1 | Cleavage |
|  |  | miR159.4 | 2.5 | -1 | Cleavage |
|  |  | miR159.5 | 2.5 | -1 | Cleavage |
|  |  | miR159.8 | 2.5 | -1 | Cleavage |
|  |  | miR159a.10 | 2.5 | -1 | Cleavage |
|  |  | miR159a.7 | 2.5 | -1 | Cleavage |
|  |  | miR159b.3 | 2.5 | -1 | Cleavage |
|  |  | miR159b.8 | 2.5 | -1 | Cleavage |
|  |  | miR159b.6 | 2.5 | -1 | Cleavage |
|  |  | miR159c.4 | 2.5 | -1 | Cleavage |
|  |  | miR159c.3 | 2.5 | -1 | Cleavage |
|  |  | miR159c.6 | 2.5 | -1 | Cleavage |
|  |  | miR159d-3p.3 | 2.5 | -1 | Cleavage |
|  |  | miR159d-3p.1 | 2.5 | -1 | Cleavage |
|  |  | miR159f.1 | 2.5 | -1 | Cleavage |
|  |  | miR159f.2 | 2.5 | -1 | Cleavage |
|  |  | miR319 | 2.5 | -1 | Cleavage |
|  |  | miR319a-3p | 2.5 | -1 | Cleavage |
|  |  | miR319a.5 | 2.5 | -1 | Cleavage |
|  |  | miR319a.3 | 2.5 | -1 | Cleavage |
|  |  | miR319b-3p | 2.5 | -1 | Cleavage |
|  |  | miR319c-3p.1 | 2.5 | -1 | Cleavage |
|  |  | miR319c.5 | 2.5 | -1 | Cleavage |
|  |  | miR319c.3 | 2.5 | -1 | Cleavage |
|  |  | miR319c.4 | 2.5 | -1 | Cleavage |
|  |  | miR319e.7 | 2.5 | -1 | Cleavage |
|  |  | miR319h | 2.5 | -1 | Cleavage |
|  |  | miR319l.2 | 2.5 | -1 | Cleavage |
|  |  | miR319l.1 | 2.5 | -1 | Cleavage |
| Growth-regulating factor 10-like | 734410 | miR396.5 | 0 | -1 | Cleavage |
| Growth-regulating factor 4-like | 759912 | miR396.5 | 0 | -1 | Cleavage |
|  | 808504 | miR396.5 | 0 | -1 | Cleavage |
|  |  | miR396.1 | 3 | -1 | Cleavage |
|  |  | miR396.3 | 3 | -1 | Cleavage |
|  |  | miR396a.3 | 3 | -1 | Cleavage |
|  |  | miR396a.1 | 3 | -1 | Cleavage |
|  |  | miR396a.2 | 3 | -1 | Cleavage |
|  |  | miR396b | 3 | -1 | Cleavage |
|  |  | miR396d.5 | 3 | -1 | Cleavage |
|  |  | miR396d.4 | 3 | -1 | Cleavage |
|  |  | miR396e-5p.2 | 3 | -1 | Cleavage |
|  |  | miR396e-5p.3 | 3 | -1 | Cleavage |
|  |  | miR396e-5p.4 | 3 | -1 | Cleavage |
|  |  | miR396e | 3 | -1 | Cleavage |
|  |  | miR396g | 3 | -1 | Cleavage |
| D-tyrosyl-tRNA(Tyr) deacylase | 760752 | miR5205b.2 | 0 | -1 | Cleavage |
|  |  | miR5205b.1 | 0 | -1 | Cleavage |
|  |  | miR5205c.1 | 0 | -1 | Cleavage |
|  |  | miR5205c.2 | 0 | -1 | Cleavage |
| Protein transport Sec1b | 732444 | miR159.4 | 1 | -1 | Cleavage |
|  |  | miR159.6 | 2 | -1 | Cleavage |
|  |  | miR159b.3 | 2 | -1 | Cleavage |
|  |  | miR159b.4 | 2 | -1 | Cleavage |
|  |  | miR159b.8 | 2 | -1 | Cleavage |
|  |  | miR159c.6 | 2 | -1 | Cleavage |
|  |  | miR159a.8 | 3 | -1 | Cleavage |
|  |  | miR159d-3p.2 | 3 | -1 | Cleavage |
|  |  | miR159d-3p.1 | 3 | -1 | Cleavage |
| Protein transport protein Sec24-like | 790208 | miR396a-3p.3 | 3 | -1 | Cleavage |
| ETHYLENE-INSENSITIVE 2 isoform X2 | 750308 | miR172c-5p | 2 | -1 | Cleavage |
| RING zinc finger and VWF domain family protein | 792638 | miR3630-3p.6 | 3 | -1 | Cleavage |
|  |  | miR3630-3p.9 | 3 | -1 | Cleavage |
|  |  | miR3630-3p.7 | 3 | -1 | Cleavage |
|  |  | miR3630-3p.5 | 2 | -1 | Cleavage |
| Leucine-rich repeat receptor-like serine/threonine-protein kinase | 802726 | miR390 | 2 | -1 | Cleavage |
|  |  | miR390a.5 | 2 | -1 | Cleavage |
|  |  | miR390a.4 | 2 | -1 | Cleavage |
|  |  | miR390a.2 | 2 | -1 | Cleavage |
|  |  | miR390a.3 | 2 | -1 | Cleavage |
|  |  | miR390c-5p | 3 | -1 | Cleavage |
| Sorting nexin 2B | 781810 | miR164a.1 | 2.5 | -1 | Cleavage |
|  |  | miR164a.2 | 2.5 | -1 | Cleavage |
|  |  | miR164b.2 | 2.5 | -1 | Cleavage |
|  |  | miR164b.3 | 2.5 | -1 | Cleavage |
|  |  | miR164b.4 | 2.5 | -1 | Cleavage |
|  |  | miR164b.5 | 2.5 | -1 | Cleavage |
|  |  | miR164e-5p | 2.5 | -1 | Cleavage |
|  |  | miR164h-5p.1 | 2.5 | -1 | Cleavage |
|  |  | miR164h-5p.2 | 2.5 | -1 | Cleavage |
| Sorting nexin-16 | 815782 | miR172c-5p | 3 | -1 | Cleavage |
| Tryptophan synthase beta chain 1 | 791620 | miR164c-3p | 2.5 | -1 | Cleavage |
| GDSL esterase/lipase | 757998 | miR167h-5p | 2.5 | -1 | Cleavage |
| DEA(D/H)-box RNA helicase family protein | 796712 | miR172d.6 | 2.5 | -1 | Cleavage |
| WD40 repeat-containing protein | 776778 | miR396a-3p.2 | 2.5 | -1 | Cleavage |
|  |  | miR396a-3p.4 | 2.5 | -1 | Cleavage |
|  |  | miR396a-3p.5 | 2.5 | -1 | Cleavage |
|  |  | miR396b-3p | 2.5 | -1 | Cleavage |
| Dynamin-related protein 3A | 760888 | miR397 | 2.5 | -1 | Cleavage |
|  |  | miR397a.1 | 2.5 | -1 | Cleavage |
|  |  | miR397a.2 | 2.5 | -1 | Cleavage |
|  | 798156 | miR397a.2 | 2.5 | -1 | Cleavage |
|  |  | miR397 | 2.5 | -1 | Cleavage |
|  |  | miR397a.1 | 2.5 | -1 | Cleavage |
| Histone-lysine N-methyltransferase | 724958 | miR477e | 2.5 | -1 | Cleavage |
| alcohol dehydrogenase | 756030 | miR156b-3p | 3 | -1 | Cleavage |
| Coiled-coil domain-containing protein | 805800 | miR156b-3p | 3 | -1 | Cleavage |
| Mechanosensitive ion channel protein 6-like | 806484 | miR156e.3 | 3 | -1 | Cleavage |
| TIME FOR COFFEE-like isoform X1 | 800780 | miR159c-5p | 3 | -1 | Cleavage |
| Receptor-like serine/threonine-protein kinase | 742978 | miR172d.6 | 3 | -1 | Cleavage |
| Citramalate synthase | 735074 | miR319a.2 | 3 | -1 | Cleavage |
| Outer membrane protein assembly factor | 800152 | miR3634-3p | 3 | -1 | Cleavage |
| Linoleate 9S-lipoxygenase 5 | 795436 | miR395i.3 | 3 | -1 | Cleavage |
|  |  | miR395i.4 | 3 | -1 | Cleavage |
|  |  | miR395i.2 | 3 | -1 | Cleavage |
| Indole-3-acetaldehyde oxidase-like isoform X2 | 793852 | miR5538.2 | 3 | -1 | Cleavage |
| Hydroxyacylglutathione hydrolase 2, mitochondrial-like isoform X1 | 815534 | miR9662a-3p | 3 | -1 | Cleavage |
| Uncharacterised protein | 694876 | miR396a.3 | 2.5 | -1 | Cleavage |
|  |  | miR396a.1 | 2.5 | -1 | Cleavage |
|  |  | miR396a.2 | 2.5 | -1 | Cleavage |
|  |  | miR396b | 2.5 | -1 | Cleavage |
|  |  | miR396e-5p.2 | 2.5 | -1 | Cleavage |
|  |  | miR396e-5p.3 | 2.5 | -1 | Cleavage |
|  |  | miR396e-5p.4 | 2.5 | -1 | Cleavage |
|  |  | miR396e | 2.5 | -1 | Cleavage |
| Uncharacterized protein | NODE_13050_length_103_cov_13.000000 | miR172d.6 | 3 | -1 | Cleavage |
| Uncharacterized protein | NODE_171799_length_206_cov_2.660194 | miR156e.3 | 3 | -1 | Cleavage |
| Hypothetical protein | 732854 | miR845a.2 | 2 | -1 | Cleavage |
| Hypothetical protein | 810388 | miR396.4 | 1.5 | -1 | Cleavage |
|  |  | miR396.6 | 1.5 | -1 | Cleavage |
|  |  | miR396.2 | 1.5 | -1 | Cleavage |
| Hypothetical protein | NODE_200464_length_391_cov_2.698210 | miR396a-3p.2 | 2.5 | -1 | Cleavage |
|  |  | miR396a-3p.4 | 2.5 | -1 | Cleavage |
