# Supplementary material for: Small RNA profiling for identification of miRNAs involved in regulation of saponins biosynthesis in Chlorophytum borivilianum
Source: BMC Plant Biol. 2017 Dec 28;17:265. doi: 10.1186/s12870-017-1214-0 (PMC5745966; doi:10.1186/s12870-017-1214-0)
Supplement: Supplementary file 8 — Saponin biosynthetic pathway specific targets from root and leaf transcriptome using miRanda. (DOCX 23 kb) [file 12870_2017_1214_MOESM8_ESM.docx]

Table: Saponin biosynthetic pathway specific targets from root and leaf transcriptome using miRanda.

| Target | Target ID | miRNA | Tot Score | Tot Energy |
| --- | --- | --- | --- | --- |
| Squalene epoxidase | 752160 | miR395h.3 | 160 | -20.9 |
|  | NODE_154767_length_925_cov_5.801081 | miR156p | 300 | -32.55 |
|  |  | miR164c-3p | 284 | -41.07 |
| Squalene synthase | NODE_73128_length_568_cov_2.860915 | miR166i-3p | 160 | -23.45 |
| Squalene monooxygenase | NODE_15652_length_879_cov_2.986348 | miR171a-3p.6 | 288 | -28.96 |
|  |  | miR168c.2 | 161 | -24.79 |
|  |  | miR168b-5p.6 | 157 | -25.21 |
|  |  | miR159.10 | 305 | -40.36 |
|  |  | miR390c-5p | 284 | -45.78 |
|  |  | miR159e.3 | 296 | -36.21 |
|  |  | miR528.7 | 158 | -21.74 |
|  |  | miR171c-3p.1 | 288 | -25.93 |
|  |  | miR159f.1 | 286 | -40.41 |
|  | NODE_73415_length_551_cov_1.722323 | miR396b | 164 | -21.09 |
| Oxidosqualene cyclase | NODE_216163_length_107_cov_2.000000 | miR477a.2 | 174 | -28.5 |
| Phosphomevalonate kinase | 782566 | miR171a-3p.6 | 162 | -20.8 |
| Mevalonate diphosphate decarboxylase | 724000 | miR319a.4 | 442 | -67.8 |
|  | NODE_156068_length_248_cov_4.000000 | miR166i.5 | 159 | -20.24 |
|  | NODE_156069_length_825_cov_4.950303 | miR156g.2 | 157 | -20.94 |
|  |  | miR156m.3 | 161 | -22.61 |
|  |  | miR156e.3 | 165 | -25.11 |
| Chloroplast 1-deoxy-d-xylulose-5-phosphate synthase | 807536 | miR172d-3p.2 | 289 | -21.77 |
| Geranyl diphosphate synthase | 736082 | miR894.6 | 284 | -23.77 |
|  | 737038 | miR396a-3p.5 | 164 | -23.88 |
|  | 764730 | miR9662a-3p | 161 | -27.17 |
|  | NODE_117321_length_638_cov_2.840125 | miR159.12 | 312 | -36.94 |
|  | NODE_54529_length_464_cov_4.943965 | miR159.10 | 305 | -35.83 |
| geranyl pyrophosphate synthase | NODE_130479_length_166_cov_3.415663 | miR894.6 | 284 | -23.77 |
| Farnesyl pyrophosphate synthase | NODE_165836_length_529_cov_5.000000 | miR172c | 309 | -38.3 |
|  | NODE_166615_length_99_cov_2.000000 | miR156m.3 | 169 | -23.86 |
|  |  | miR156e.3 | 173 | -25.88 |
|  |  | miR156g.2 | 165 | -21.31 |
| 4-Hydroxy-3-methylbut-2-enyl diphosphate reductase | 713070 | miR172c-5p | 299 | -32.28 |
|  | 757110 | miR156j.2 | 156 | -24.52 |
|  |  | miR156a.1 | 161 | -20.4 |
|  |  | miR1425-5p | 170 | -25.49 |
|  | 780586 | miR398a-3p.5 | 157 | -21.4 |
|  |  | miR164b-3p | 171 | -25.55 |
| 4-Hydroxy-3-methylbut-2-en-1-yl diphosphate synthase | NODE_115055_length_2136_cov_6.770599 | miR164b.5 | 286 | -39.72 |
|  |  | miR164c-5p | 286 | 125.65 |
|  |  | miR164b.4 | 286 | -40.95 |
|  |  | miR164b.3 | 286 | -37.59 |
|  |  | miR528.7 | 308 | -51.2 |
|  |  | miR159.10 | 424 | -54.58 |
|  |  | miR319e.12 | 315 | -41.94 |
|  |  | miR164a.2 | 286 | -37.44 |
| 4-Hydroxy-3-methylbut-2-enyl diphosphate reductase | NODE_169967_length_383_cov_3.000000 | miR156p | 287 | -31.16 |
| Hydroxymethylbutenyl diphosphate reductase | NODE_169971_length_254_cov_8.000000 | miR477e | 157 | -20.62 |
| Hydroxymethylglutaryl-CoA synthase | NODE_215584_length_494_cov_2.872470 | miR167c.4 | 158 | -23.06 |
|  |  | miR159e.3 | 288 | -32.78 |
| Cycloartenol synthase | NODE_194214_length_665_cov_2.651128 | miR319e.12 | 158 | -21.8 |
|  |  | miR159.10 | 286 | -47.91 |
|  | NODE_49589_length_645_cov_2.852713 | miR1425-5p | 286 | -37.63 |
| epoxide hydrolase A | NODE_162885_length_210_cov_5.280952 |  | 163 | -21.88 |
| Glutathione S-transferase-1 | NODE_178862_length_74_cov_12.000000 | cbo-miR5 | 156 | -23.61 |
| Isopentenyl diphosphate isomerase 2 | CL1275Contig1 | miR167g-5p | 165 | -20.29 |
|  |  | miR167g.3 | 165 | -22.46 |
|  |  | miR167c.11 | 165 | -20.78 |
|  |  | miR167c.4 | 165 | -20.29 |
|  |  | miR167c.10 | 165 | -21.43 |
|  |  | miR167c.8 | 165 | -22.77 |
|  |  | miR167f-5p.2 | 165 | -21.67 |
| Transketolase | NODE_91280_length_84_cov_4.000000 | cbo-miR3 | 158 | -26.9 |
| UDP-glycosyltransferase-like protein | NODE_208057_length_166_cov_3.222892 | miR159.10 | 170 | -22.63 |
| UDP-glycosyltransferase-like protein | NODE_182700_length_140_cov_3.000000 | miR477e | 157 | -24.42 |
| UDP-glycosyltransferase 73B3-like | NODE_148079_length_685_cov_6.000000 | miR166g-3p.3 | 159 | -21.63 |
| UDP-glycosyltransferase 73C3-like | NODE_114532_length_195_cov_3.938462 | miR164a.2 | 300 | -46.95 |
|  |  | miR164b.4 | 300 | -47.97 |
|  |  | miR164b.3 | 296 | -43.84 |
|  |  | miR164b.5 | 300 | -46.99 |
|  |  | miR159.10 | 164 | -24.05 |
| UDP-glycosyltransferase 74B1-like | CL1431Contig1 | miR156e.2 | 160 | -23.45 |
| UDP-glycosyltransferase 85B2 | NODE_152453_length_117_cov_10.000000 | miR477e | 158 | -22.35 |
|  | NODE_152459_length_117_cov_3.000000 | miR477e | 158 | -22.35 |
| UDP-glycosyltransferase 85A1-like | NODE_29197_length_355_cov_3.876056 | miR156f.4 | 160 | -22 |
|  |  | miR408b-3p | 280 | -25.07 |
| UDP-glycosyltransferase 85A1 | NODE_43550_length_898_cov_2.660356 | miR395b.2 | 171 | -22.04 |
|  |  | miR395h.3 | 175 | -23.54 |
|  |  | miR395h.4 | 175 | -23.54 |
|  |  | miR395i.4 | 175 | -22.38 |
|  |  | miR395b.3 | 175 | -22.38 |
| UDP-glycosyltransferase 85A2 isoform 1 | NODE_185858_length_789_cov_2.859316 | miR319c-5p | 283 | -23.34 |
|  |  | miR319a-5p.4 | 283 | -24.85 |
| UDP-glycosyltransferase 85A2-like | NODE_200163_length_467_cov_2.207709 | miR390a.5 | 157 | -21.97 |
|  |  | miR390c-5p | 157 | -25.62 |
| UDP-glycosyltransferase 87A1-like | NODE_165286_length_88_cov_2.000000 | miR535b.5 | 170 | -25.76 |
| UDP-glycosyltransferase 88A1-like isoform 1 | NODE_216956_length_80_cov_1.950000 | miR166k-3p.4 | 159 | -21.13 |
|  |  | miR168a.3 | 159 | -23.05 |
| UDP-glycosyltransferase 91A1-like | NODE_183807_length_273_cov_2.864469 | miR168b-5p.6 | 283 | -46.02 |
| UDP-glycosyltransferase 92A1-like | NODE_107660_length_615_cov_2.718699 | miR477e | 295 | -38.91 |
| Cytochrome P450 family protein | NODE_153751_length_1144_cov_6.000000 |  | 298 | -39.44 |
| cytochrome P-450 | NODE_123550_length_459_cov_3.755991 | cbo-miR1 | 157 | -20.39 |
| cytochrome P450 89A2-like | NODE_17323_length_306_cov_10.294118 |  | 158 | -21.8 |
| Cytochrome p450 90b1-like | 813156 | miR156f.4 | 156 | -20.17 |
|  |  | miR156e-5p | 291 | -33.77 |
|  |  | miR396e-5p.4 | 289 | -34.11 |
|  |  | miR167g.3 | 289 | -34.11 |
| cytochrome P450 94A1-like | NODE_125924_length_278_cov_2.352518 |  | 157 | -24.51 |
| beta-glucosidase 12-like isoform 1 | 815726 | cbo-miR1 | 297 | -30.86 |
