# Supplementary material for: Small RNA profiling for identification of miRNAs involved in regulation of saponins biosynthesis in Chlorophytum borivilianum
Source: BMC Plant Biol. 2017 Dec 28;17:265. doi: 10.1186/s12870-017-1214-0 (PMC5745966; doi:10.1186/s12870-017-1214-0)
Supplement: Supplementary file 9 — List of miRNAs involved in regulating both primary and secondary metabolism. (DOCX 15 kb) [file 12870_2017_1214_MOESM9_ESM.docx]

Table: List of miRNAs involved in both primary and secondary metabolism.

| **miRNA** | **Target involved in secondary metabolism** | **Targets other than Secondary metabolism** |
| --- | --- | --- |
| miR395h.3 | Squalene epoxidase,  UDP-glycosyltransferase 85A1 | Low affinity sulfate transporter 3 |
| miR156p | Squalene epoxidase,  4-Hydroxy-3-methylbut-2-enyl diphosphate reductase | SPL7, SPL12, SPL16 |
| miR164c-3p | Squalene epoxidase | Tryptophan synthase beta chain 1 |
| miR171a-3p.6 | Squalene monooxygenase | Scarecrow-like protein 6 |
| miR168b-5p.6 | Squalene monooxygenase, UDP-glycosyltransferase 91A1-like |  |
| miR159.10 | Squalene monooxygenase, Geranyl diphosphate synthase, 4-Hydroxy-3-methylbut-2-en-1-yl diphosphate synthase, Cycloartenol synthase, UDP-glycosyltransferase-like protein | Protein toll, synaptotagmin-2-like, Transcription factor GAMYB isoform X1 |
| miR390c-5p | Squalene monooxygenase,  UDP-glycosyltransferase 85A2-like | Leucine-rich repeat receptor-like serine/threonine-protein kinase |
| miR159e.3 | Squalene monooxygenase, Hydroxymethylglutaryl-CoA synthase | Protein toll |
| miR171c-3p.1 | Squalene monooxygenase | Scarecrow-like protein 6 |
| miR159f.1 | Squalene monooxygenase | Protein toll, Transcription factor GAMYB isoform X1 |
| miR396b | Squalene monooxygenase | Growth-regulating factor 4-like |
| miR477a.2 | Oxidosqualene cyclase | Signal transduction histidine kinase |
| miR319a.4 | Mevalonate diphosphate decarboxylase | UDP-N-acetylglucosamine diphosphorylase, Transcription factor GAMYB isoform X1 |
| miR156e.3, miR156m.3, miR156g.2 | Mevalonate diphosphate decarboxylase, Farnesyl pyrophosphate synthase | SPL7, SPL12, SPL16, Mechanosensitive ion channel protein 6-like |
| miR172d-3p.2 | Chloroplast 1-deoxy-d-xylulose-5-phosphate synthase | APETALA2-like protein, ABC1 protein |
| miR396a-3p.5 | Geranyl diphosphate synthase | WD40 repeat-containing protein |
| miR9662a-3p | Geranyl diphosphate synthase | Hydroxyacylglutathione hydrolase 2, mitochondrial-like isoform X1 |
| miR159.12 | Geranyl diphosphate synthase | TRIGALACTOSYLDIACYLGLYCEROL 3, chloroplastic, Protein toll |
| miR172c | Farnesyl pyrophosphate synthase | APETALA2-like protein, Sorting nexin-16 |
| miR172c-5p | 4-Hydroxy-3-methylbut-2-enyl diphosphate reductase | ETHYLENE-INSENSITIVE 2 isoform X2 |
| miR156j.2 | 4-Hydroxy-3-methylbut-2-enyl diphosphate reductase | SPL7, SPL12, SPL16 |
| miR156a.1 | 4-Hydroxy-3-methylbut-2-enyl diphosphate reductase | SPL7, SPL12, SPL16 |
| miR164b.5 | 4-Hydroxy-3-methylbut-2-en-1-yl diphosphate synthase, UDP-glycosyltransferase 73C3-like | NAC domain-containing protein 21/22-like, UDP-glucuronic acid decarboxylase 6-like, Sorting nexin 2B |
| miR164c-5p | 4-Hydroxy-3-methylbut-2-en-1-yl diphosphate synthase | NAC domain-containing protein 21/22-like, UDP-glucuronic acid decarboxylase 6-like |
| miR164b.4, miR164b.3, miR164a.2 | 4-Hydroxy-3-methylbut-2-en-1-yl diphosphate synthase, UDP-glycosyltransferase 73C3-like | NAC domain-containing protein 21/22-like, UDP-glucuronic acid decarboxylase 6-like, Sorting nexin 2B |
| miR319e.12 | 4-Hydroxy-3-methylbut-2-en-1-yl diphosphate synthase, Cycloartenol synthase | Protein toll, Transcription factor GAMYB isoform X1 |
| miR477e | Hydroxymethylbutenyl diphosphate reductase, UDP-glycosyltransferase-like protein | Signal transduction histidine kinase, Mitogen-activated protein kinase 4, Histone-lysine N-methyltransferase |
| miR167c.4 | Hydroxymethylglutaryl-CoA synthase, Isopentenyl diphosphate isomerase 5 | xyloglucan endotransglucosylase/hydrolase protein 24-like |
| miR167g-5p | Isopentenyl diphosphate isomerase 2 | xyloglucan endotransglucosylase/hydrolase protein 24-like |
| miR167g.3 | Isopentenyl diphosphate isomerase 3, Cytochrome p450 90b1-like | xyloglucan endotransglucosylase/hydrolase protein 24-like |
| miR167c.11 | Isopentenyl diphosphate isomerase 4 | xyloglucan endotransglucosylase/hydrolase protein 24-like |
| miR167c.10 | Isopentenyl diphosphate isomerase 6 | xyloglucan endotransglucosylase/hydrolase protein 24-like |
| miR167c.8 | Isopentenyl diphosphate isomerase 7 | xyloglucan endotransglucosylase/hydrolase protein 24-like |
| miR167f-5p.2 | Isopentenyl diphosphate isomerase 8 | xyloglucan endotransglucosylase/hydrolase protein 24-like |
| miR166g-3p.3 | UDP-glycosyltransferase 73B3-like | HD-Zip protein ATHB-31, HD-Zip protein ATHB-23, Class III homeobox-leucine zipper protein |
| miR156e.2 | UDP-glycosyltransferase 74B1-like | SPL7, SPL12, SPL16, L-type lectin-domain containing receptor kinase |
| miR156f.4 | UDP-glycosyltransferase 85A1-like, Cytochrome p450 90b1-like | SPL7, SPL12, SPL16 |
| miR395b.2, miR395h.4 | UDP-glycosyltransferase 85A1 | Low affinity sulfate transporter 3 |
| miR395i.4 | UDP-glycosyltransferase 85A1 | Low affinity sulfate transporter 3, Linoleate 9S-lipoxygenase 5 |
| miR395b.3 | UDP-glycosyltransferase 85A1 | Low affinity sulfate transporter 3 |
| miR390a.5 | UDP-glycosyltransferase 85A2-like | Leucine-rich repeat receptor-like serine/threonine-protein kinase |
| miR156e-5p | Cytochrome p450 90b1-like | SPL7, SPL12, SPL16 |
| miR396e-5p.4 | Cytochrome p450 90b1-like | Growth-regulating factor 4-like |
